# Assessing spacer acquisition rates in *E. coli* type I-E CRISPR arrays

**DOI:** 10.3389/fmicb.2024.1498959

**Published:** 2025-01-20

**Authors:** Luke J. Peach, Haoyun Zhang, Brian P. Weaver, James Q. Boedicker

**Affiliations:** ^1^Department of Biological Sciences, University of Southern California, Los Angeles, CA, United States; ^2^Department of Physics and Astronomy, University of Southern California, Los Angeles, CA, United States

**Keywords:** CRISPR adaptation, type I-E CRISPR, Cas1–Cas2, array expansion, spacer acquisition rates, non-homologous end joining, CRISPR interference, phage infection defense

## Abstract

CRISPR/Cas is an adaptive defense mechanism protecting prokaryotes from viruses and other potentially harmful genetic elements. Through an adaptation process, short “spacer” sequences, captured from these elements and incorporated into a CRISPR array, provide target specificity for the immune response. CRISPR arrays and array expansion are also central to many emerging biotechnologies. The rates at which spacers integrate into native arrays within bacterial populations have not been quantified. Here, we measure naïve spacer acquisition rates in *Escherichia coli* Type I-E CRISPR, identify factors that affect these rates, and model this process fundamental to CRISPR/Cas defense. Prolonged Cas1–Cas2 expression produced fewer new spacers per cell on average than predicted by the model. Subsequent experiments revealed that this was due to a mean fitness reduction linked to array-expanded populations. In addition, the expression of heterologous non-homologous end-joining DNA-repair genes was found to augment spacer acquisition rates, translating to enhanced phage infection defense. Together, these results demonstrate the impact of intracellular factors that modulate spacer acquisition and identify an intrinsic fitness effect associated with array-expanded populations.

## Introduction

**Figure 1:**
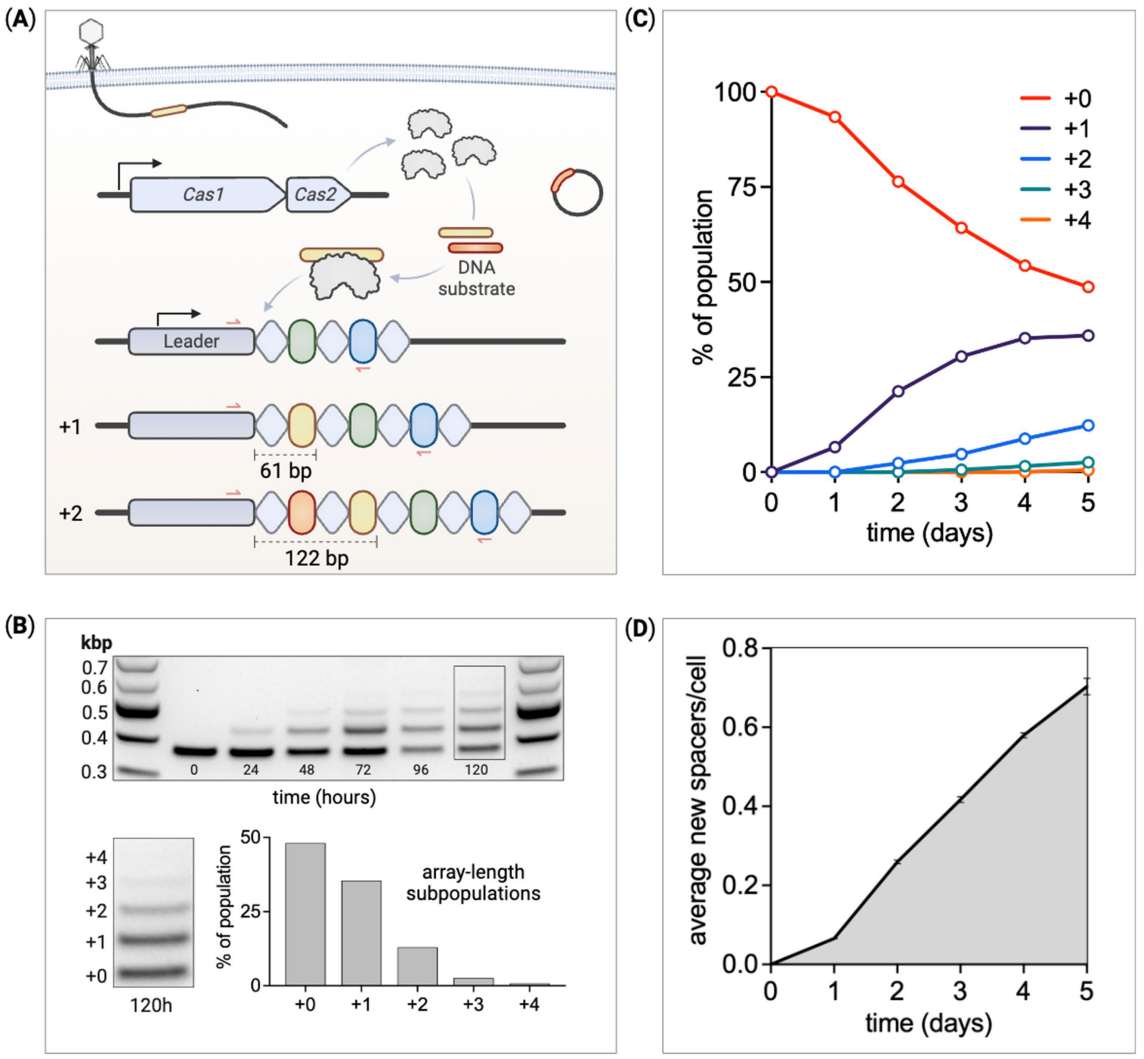
Quantifying the temporal dynamics of spacer acquisition. **(A)** An overview of the adaptation phase of CRISPR adaptive immunity in the *E. coli* Type I-E system. CRISPR arrays are made up of alternating repeats (diamonds) and spacers (ovals) along with an upstream leader sequence. Cas1 and Cas2 form a six-subunit complex that captures and processes small fragments of DNA before integration as spacers between the leader and first repeat. Each spacer integration duplicates the leader proximal repeat, together expanding the array by 61 base pairs. Red arrows represent PCR primer binding sites used to detect array expansion. **(B)** PCR and DNA gel electrophoresis measure changes in array lengths within a culture of cells expressing Cas1–Cas2. At 120 h, cells within the population have gained up to four new spacers. The band intensity is used to quantify the relative proportion of cells at each array length. **(C)** The ratio of cells at each array length is tracked over several days. **(D)** The average number of new spacers acquired per cell within a culture is calculated at each time point through the experiment.

CRISPR/Cas defense enables adaptive invader targeting through an updating array of clustered regularly interspaced short palindromic repeats (CRISPR) containing a repository of immunological targets (spacers) stored in the host chromosome. Arrays are expressed and processed into short RNA sequences (crRNA) that guide CRISPR-associated (Cas) effectors to eliminate targets with crRNA complementarity (Barrangou et al., 2007; Garneau et al., 2010; Marraffini, 2015). Upon infection, the CRISPR/Cas immune response begins with an adaptation phase whereby a small fraction of infected cells incorporates invader-derived spacers between repeat sequences within an array (Figure 1A). Acquisition of spacers from sources not previously encountered or in the absence of Cas effector machinery is referred to as naïve spacer acquisition (Fineran and Charpentier, 2012).

*In vivo* spacer acquisition studies often utilize plasmid-based arrays and deep sequencing to identify newly integrated spacer sequences. These studies have been crucial for expanding our fundamental understanding of CRISPR adaptation and for the development of new applications by providing insight into the relative differences in acquisition frequencies of specific spacer sequences (Heler et al., 2019; Sheth et al., 2017), yet rates at which new spacers are integrated into native arrays have not been rigorously studied. Although many mechanistic details of spacer acquisition have been reported (Arslan et al., 2014; Ivančić-Baće et al., 2015; McGinn and Marraffini, 2016, 2019; Nuñez et al., 2015), the temporal dynamics of this process and how these dynamics are modulated by cellular parameters are understudied. Several promising spacer recording applications are being developed that may benefit from a broader understanding of naïve acquisition and a simple method to detect rate changes. These include recording intra-and extracellular biological events within a lineage of cells over time (Munck et al., 2020; Sheth and Wang, 2018), long-term ordered recording of transcriptional events (Lear and Shipman, 2023), and digital-to-biological data storage (Shipman et al., 2017; Yim et al., 2021). In this study, naïve spacer acquisition rates are quantified for *Escherichia coli* Type I-E CRISPR (Koonin et al., 2017). We calculate mean spacer acquisition rates per cell and identify intracellular factors that modulate these rates.

Spacer integrations are carried out by the Cas1–Cas2 integrase complex. This process not only immunizes the host but generates a heritable and chronological memory bank of infection history (Amitai and Sorek, 2016; Jackson et al., 2017; Sternberg et al., 2016). CRISPR arrays identified in wild-type bacterial genomes contain up to a few 100 spacers (Martynov et al., 2017; Pourcel et al., 2020). The size of an array repertoire is optimized to maintain the diversity proportional to the environmental threat, while being small enough to avoid diluting interference machinery with obsolete spacers. Spacers are derived from sequences that contain a protospacer adjacent motif (PAM), a short sequence that differentiates the array spacer from the protospacer target (Wang et al., 2015). Spacer integrations are polarized, generally occurring at the leader end of the array (Bernick et al., 2012). Directly upstream of the array, the leader sequence contains the CRISPR promoter and segments required for spacer integration (Díez-Villaseñor et al., 2013; Mitić et al., 2023; Wei et al., 2015). The leader proximal repeat is duplicated with each spacer addition resulting in array expansion, the combined length of these two elements (Jackson et al., 2017; Yosef et al., 2012). Spacer integrations in *E. coli* Type I-E CRISPR usually expand the array by 61 base pairs (33-bp spacers; 28-bp repeats) (Shipman et al., 2016). In this system, arrays are expressed as precursor crRNA and subsequently processed into mature crRNA by the CRISPR-associated complex for antiviral defense, known as Cascade. Each crRNA is made up of a spacer and part of each adjacent repeat. Cascade is guided by crRNA to a target sequence (protospacer) complementary to the spacer-derived region within the crRNA. Once bound to a target, Cascade recruits helicase–nuclease Cas3 to degrade the DNA (He et al., 2020; Liu and Doudna, 2020; Mulepati and Bailey, 2011; Yoshimi et al., 2022). This defense strategy enables adaptive invader targeting by updating the array as foreign DNA is encountered over time (Bolotin et al., 2005; Mojica et al., 2005; Pourcel et al., 2005).

Several studies have quantified spacer acquisition under laboratory conditions. Genomic array deep-sequencing data have been used to quantify array-expanded fractions from Cas1–Cas2 expressing cultures at a single time point post-induction (Levy et al., 2015); however, this provides limited insight into acquisition rates. PCR amplifications using primers flanking the leader-repeat1 integration site produce amplicon band intensities with ratios proportional to the expanded-array subpopulations. This assay was used to accurately measure expanded fractions from a CRISPR-adapted culture (Yosef et al., 2023), but also at a single time point post-induction. Plasmid barcoding has been utilized to identify independent acquisition events in bacterial cultures to characterize relative rates of spacer acquisition (Heler et al., 2017). This method can provide accurate acquisition rate comparisons between strains but does not elucidate the extent of acquisition per cell in bacterial cultures.

In this study, strains of *E. coli* were engineered for controlled expression of Cas genes to quantify CRISPR-array spacer acquisition dynamics. PCR and DNA gel electrophoresis were utilized to measure the extent of spacer acquisition in genomic CRISPR arrays within bacterial cultures over multi-day serial passage experiments. By tracking array expansion within populations of *E. coli*, rates of spacer acquisition were calculated. We modified several intracellular parameters and quantified their respective impacts on spacer acquisition rates. These included Cas1–Cas2 expression levels, the presence of a high copy number plasmid, the presence of multiple CRISPR arrays in the genome, and the expression of heterologous non-homologous end-joining (NHEJ) genes from *Mycobacterium smegmatis*. NHEJ expression significantly enhanced spacer acquisition rates, with this increased CRISPR adaptation providing greater phage infection defense. In modeling spacer acquisition from the array expansion data, it appeared that spacer acquisition slowed for populations of cells with longer arrays. Model parameterization identified reduced fitness associated with array-expanded populations as the likely cause, which was subsequently supported with competition experiments.

## Materials and methods

### Bacterial strains and growth conditions

All bacterial strains in this study were derived from *E. coli* BL21-AI. Strains were cultivated in Luria-Bertani (LB) media at 37°C with 320 rpm shaking. Axenic cultures were maintained by dosing with 50 μg/mL spectinomycin as all strains contained a constitutive, genomic spectinomycin resistance marker. Where appropriate, other antibiotics, such as carbenicillin (100 μg/mL), kanamycin (50 μg/mL), gentamicin (15 μg/mL), and chloramphenicol (25 μg/mL), were used. Cultures subjected to phage infection were grown in LB media also supplemented with 0.2% maltose and 10 mM MgSO_4_ to facilitate phage adsorption. A list of strains used in this study can be found in Supplementary Table S1.

### Strain construction

Two primary parental strains were used in this study: (a) a CRISPR adaptation (Cas1–Cas2) enabled base recording strain, containing no interference (Cascade–Cas3) machinery, (b) a host with both CRISPR adaptation and interference machinery. T7-lac-Cas1-Cas2 was genome integrated along with a spectinomycin resistance marker using a previously described Lambda red recombineering method (Sharan et al., 2009). Other constructs (RhaB-Cascade-Cas3, NHEJ, mini-array) were genome integrated using the markerless guide-RNA-assisted targeting system INTEGRATE (Vo et al., 2021). Briefly, the INTEGRATE method consists of a single plasmid assembly to program a defined cargo of interest (up to ~10 kbp) and a spacer specifying the genomic target for cargo integration. Gibson Assembly was used to produce these cargo (e.g., RhaB–Cascade–Cas3) plasmids intended for genome integration. Spacers were programmed into the plasmid by first performing a BsaI restriction enzyme digestion followed by 32-bp spacer ligation into the INTEGRATE array. The plasmid contains a temperature-sensitive origin of replication for plasmid curing at 41°C after cargo integration. Genomic sites of integration were chosen based on previous reports (Park et al., 2020). Genome-integrated sequences are listed in Supplementary material Text S3. A list of plasmids used in this study can be found in Supplementary Table S2.

### Spacer acquisition detection and quantification

Spacers are integrated into *E. coli* Type I-E CRISPR arrays at the leader–repeat1 junction. We used PCR primers flanking this site to identify array expansion in both clonal and mixed cultures. A list of primers used in this study can be found in Supplementary Table S3. The parental BL21-AI host strain contains 13 native spacers in the array. We tracked expansion as any new spacers incorporated into the array (e.g., 14 total spacers is +1; 15 is +2). The PCR primers used to detect array expansion annealed to the leader sequence (FP) and native spacer-5 (RP), capturing the site of integration. The unexpanded, +0 parental amplicon is 379 bp, and each new spacer expands the amplicon by 61 bp (e.g., +1 = 440 bp; +2 = 501 bp). To detect expansion from liquid cultures, 15 μL of the culture was mixed in a PCR tube with 15 μL of water. The tubes were placed in a thermal cycler at 95°C for 15 min to generate a genomic template for PCRs. From colonies, biomass was scraped with a pipette tip and mixed with 15 μL of water prior to running the same thermal step. The percentage of the total population at each array length was calculated; therefore, small variations in the number of cells used as PCR templates would not influence the results; 25 μL of PCRs were run with 21 amplification cycles using NEB OneTaq DNA polymerase and 5 μL of template. After the PCRs, samples were run on electrophoresis gels to separate the amplicons by size; 20 μL of each sample and 7 μL of gel loading dye (no SDS) were mixed, with 20 μL of this mix run on 2% agarose TBE gels; 8 μL of the 1 kb-plus DNA ladder was run in the first and last lanes, with the average ladder band intensities from the two lanes used for subsequent calculations. Gels were run at 110 volts for 70 min to achieve adequate band separation for individual quantifications. Gels were imaged on a Bio-Rad Gel Doc EZ Imager, with the images imported into quantification software (GelAnalyzer) for further analysis. Amplicon band intensities were measured using image-pixel analysis. Band intensities were converted to picomolar concentrations using the ladder bands. With the two ladder bands closest in size to experimental bands, pmols/intensity values were used to convert intensity values to pmol concentrations in each detectable band.

### Spacer acquisition time course experiment

Spacer acquisition rates were characterized over a 5-day time course with constant Cas1–Cas2 induction (0.05 mM IPTG, 0.2% w/w arabinose), sampling every 24 h and quantifying the resulting PCR bands (Supplementary Figure S1). An SDS-PAGE protein gel shows the Cas1 band present with induction and absent without, for expression from both a plasmid and the genome (Supplementary Figure S2). In a separate set of experiments, the base recording strain with the genome integrated Cas1–Cas2 operon was induced with 0.05 mM IPTG and 0.2% arabinose prior to total cell lysate harvesting at several time points post-induction (Supplementary Figure S3A). Cultures of the same strain dosed with fixed arabinose (0.2%) and variable IPTG (0–5 mM) were harvested at 3 h post-induction for total cell lysate SDS-PAGE analysis via Cas1 band intensity quantification (Supplementary Figure S3B). The Cas1 bands were quantified using GelAnalyzer software and normalized to the housekeeping protein GAPDH (Supplementary Figures S3C,D).

To start each time course, overnight cultures were normalized to OD600 with 15uL used to inoculate 3 mL of fresh media in 14-mL Falcon tubes. Samples were taken from the overnight cultures for array (leader proximal) PCR to establish time-0 amplicon band proportions prior to induction. For a given culture, six PCRs were performed over the time course, once each day. At the end of the experiment, these samples were run together on the same 8-well DNA electrophoresis gel. PCR amplicon bands were quantified and converted to pmols. Validation of this quantification method is shown in Supplementary Figure S4. Samples from each culture were cryopreserved at the end of each time course for further analysis as needed.

### Fitting procedure for CRISPR array expansion rates

Model parameters were fit to the mean of the experimental replicates. For the initial calculation of the array expansion rate reported, the loss of cells at array length +0 was fit to Equation 5 using the MATLAB Curve Fitting Tool with residuals weighted by the inverse of the standard deviation. To account for the fitness effect of array expansion, array expansion rates reported were generated by solving Equations 3, 4, 7. Data were fit from 24 h to the end of experiment. Optimal parameter values were determined using a weighted least squares fit, implemented with inbuilt lsqnonlin fitting function of MATLAB (trust region reflective algorithm). Weights for a given data point were defined as the inverse of the standard deviation at that data point. In cases where no band was detected experimentally, the residual was assigned a weight of 0. For the case of the strain with two arrays, the equations were modified to account for fitness costs associated with expanding both arrays simultaneously, see Supplementary material Text S2. Estimations of error in fit parameters were calculated by performing bootstrapping on each data set. Parameters and errors reported in this study result from averaging 500 bootstrapping iterations. For statistical comparisons of expansion rates, see Supplementary Table S4.

### Expanded-array sequencing

Clones were isolated by plating diluted cultures onto LB agar after 1-day or 5-day induction time course experiments. PCR amplicons were generated with the method previously described to screen for array-expanded colonies. For expanded clones that were sent for sequencing, a second PCR was performed, and subsequent PCR cleanup was carried out for each post-PCR sample. Sanger sequencing was performed on these clonal samples using one of the two standard array-PCR primers (Supplementary Table S5). Sequencing data were imported into SnapGene for amplicon analysis to identify the newly integrated spacers.

### Simulations

In these simulations, the initial population contained 9,780 cells with array length +0 and 220 cells with array length +1, based on experimental measurements of the composition 24h post induction. The simulation had a time step of 5 min with an end time of 10 days. For the initial model, the culture grew exponentially with a growth rate constant of 0.02 1/min. Upon reaching a population size of 10^8^ cells, 10^4^ cells were selected at random to inoculate a new culture. At each timepoint, each cell had a probability of 8.16833 × 10^−5^ of gaining one new spacer. Simulations were modified to incorporate a reduction in the array expansion rate, reduction in the growth rate, or mutations. For simulations with mutations, each cell had a low probability (10^−5^ to 10^−7^) of becoming a mutant with an array expansion rate of 0 and a variable gain of fitness (either +0% or +3%).

### Competition experiments

For the base recording strain with and without pUC19, the standard Cas1–Cas2 induction experiment was carried out for 24 h. At the 24 h mark cells were passaged 1:100 into fresh LB media with antibiotics but without induction chemicals (IPTG and arabinose). From this point on, cultures were not exposed to IPTG or arabinose. Cultures were grown from 24 to 32 h to allow for residual Cas1–Cas2 to degrade. At 32 h, each culture was sampled for PCR analysis across the spacer integration site to quantify baseline (expanded cells)/(all cells) population ratios. Cultures were again sampled for PCR experiments and passaged 1:100 at 48 h and 72 h, with the last PCR samples run at 96 h. The PCR ratios were quantified at each time point to assess changes in relative proportions over time (Supplementary Figures S5A,B). From one of the three replicates of the base strain w/pUC19, the culture from the 32-h time point was plated to single cells onto LB agar. Hundred clonal colonies were PCR screened across the array integration site; 14 of the 100 clones contained an expanded array (all +1). These +1 clones were individually competed against the parental +0. In these 14 clonal competition experiments, overnight cultures were OD600 normalized and 50:50 volumes of the +1 clone and +0 were first mixed into a sterile 1.5-mL Eppendorf tube. This mixture was used as PCR template for the 0 time point and used to seed the initial 3 mL of cultures (30 μL) containing antibiotics. No induction chemicals were used. Competition experiments were run for 48 h with passaging occurring at 24 h and PCRs run on samples at 0 h, 24 h, and 48 h (Supplementary Figures S5C–E). Carbenicillin and spectinomycin antibiotics were dosed into all 14 cultures except for two. Slow-growing clones 8 and 14 were sensitive to spectinomycin so only carbenicillin was used for those two competition experiments.

### Phage propagation

An *E. coli* lysogen containing bacteriophage Lambda prophage was used to produce purified phage for our infection experiments. For isolation of bacteriophage Lambda, an engineered strain of *E. coli* containing plasmid pB33recA730 allows for induction of the lytic cycle with arabinose. An overnight culture of this strain was passaged 1:200 into 3 mL of fresh media with chloramphenicol. The culture was grown until it reached OD600 ~ 0.4, at which point it was dosed with arabinose at a final concentration of 0.2%. The culture was protected from light and grown at 37°C until lysis occurred, and the culture became clear. The solution was then centrifuged to clear the debris. Supernatant was transferred to a fresh tube, and chloroform was added to sterilize (100 μL chloroform for 5–10 mL supernatant). The solution was transferred to a polystyrene tube to extract the chloroform before sample transfer to a 15-mL conical tube and wrapped in tinfoil for storage at 4°C.

### Plaque formation assay

A 10-fold dilution series was made from purified bacteriophage. The 10^6^, 10^7^, and 10^8^ dilutions were separately plated with MG1655 *E. coli* suspended in 0.7% top agar containing LB supplemented with maltose and MgSO_4_. The plates were incubated overnight at 37°C and the resulting plaques enumerated to determine the purified-phage concentration.

### Bacteriophage infection assay

*Escherichia coli* strains were inoculated and cultured overnight at 37°C in LB media supplemented with maltose and MgSO_4_ (LBMM); 3 mL of fresh LBMM was prepared in 14-mL falcon tubes along with the appropriate antibiotics and induction chemicals to express Cas1–Cas2 (IPTG, arabinose), Cascade, and Cas3 (rhamnose). Overnight cultures were normalized to OD600 prior to 15 μL inoculations with or without phage. To induce infection, Lambda phage was inoculated at a multiplicity of infection (MOI) of 0.02. Immediately after inoculation, these 3 mL of cultures were distributed as 200 μL replicates into a flat-bottom 96-well plate (Corning). Absorbance at 600 nm was recorded every 20 min for 21 h using a microplate reader (TECAN Infinite 200 PRO). For each strain, uninfected and infected OD600 was plotted over the course of the experiment and area under the curve, using the trapezoidal rule, was quantified to calculate the percentage of growth inhibition (PI). This is calculated by finding the difference in areas under the curve for uninfected control and infected cultures (45). The areas are calculated from a start point of detection (SPD) to an end point of detection (EPD). The SPD is the threshold at which growth is first detected in the cultures, defined as when the uninfected control reaches a growth rate of 0.001 OD units per minute. The EPD was defined as 15 h post SPD. The PI values were analyzed to approximate the relative phage resistance for each *E. coli* strain, see Supplementary Table S4 for statistical tests. Doubling times (Td) were calculated from the uninfected cultures for each strain (Supplementary Figure S6).

## Results

To monitor CRISPR array expansion over time, PCR was used to measure the proportion of the array-expanded populations at each array length. This assay has been reported previously to identify array expansion after culturing cells for several hours with Cas1–Cas2 induction (Wei et al., 2015). We used *E. coli* BL21-AI as our host strain to study spacer acquisition (Wei et al., 2015; Yosef et al., 2012). This strain is deficient in all Type I-E Cas components but does include a native CRISPR array. We integrated an inducible Cas1–Cas2 operon into the genome. This allowed us to study spacer acquisition in a “base recording strain” free of Cas interference machinery (Cascade/Cas3) and plasmids. We did, however, transform pUC19 into some spacer recording strains as it provides excess Cas1–Cas2 template to amplify acquisition. The parental BL21-AI CRISPR array contains 13 conserved spacers and 14 repeats. PCR primers flanking the leader-proximal end of the array were used to amplify samples from cultures induced for Cas1–Cas2 expression (Figure 1A). One of the primers is complementary to part of the upstream leader sequence and the other to conserved spacer-5. Unexpanded parental arrays produce 379 base pair amplicons with expanded subpopulations 61 base pairs longer for each new spacer addition. PCR products were separated by size on agarose gels through DNA electrophoresis allowing us to differentiate band intensities (Figure 1B). These amplicon bands were converted to picomoles and subsequently used to evaluate array-length subpopulation ratio changes throughout our experiments. Control experiments verified that this method can accurately measure array lengths that represent as little as ~0.5% of the population with an error of approximately 3.6% (Supplementary Figures S7C,D), consistent with previously reported data (Amlinger et al., 2017).

Over time, Cas1–Cas2 induction produces longer arrays within the population due to the continued addition of new spacers (Figure 1B). Cultures induced for constant Cas1–Cas2 expression were grown for 5 days, with subculturing and PCR-based length measurements performed every 24 h. Amplicon bands were sufficiently separated via gel electrophoresis, with band intensities proportional to the frequency of that array length within the population. The DNA ladder with bands of known concentrations were used to convert experimental band intensities to pmols. The fraction of cells at each array length, $f_{i}$, can be calculated using Equation 1,


(1) $$f_{i}=N_{i}/\sum N_{i},$$

where $N_{i}$ is the number of cells with array length i. Cells were cultured over 120 h, with unexpanded parental arrays gradually decreasing as expanded subpopulations increased in proportion (Figure 1C). Given the percentage of the population at each detectable array length, the average array length can be calculated using Equation 2,


(2) $$\bar{L}=\sum f_{i}L_{i},$$

where $L_{i}$ is the array length of subpopulation i and $\bar{L}$ is the average array length across the whole population. The extent of expansion in the population, calculated as the “average new spacers per cell” over time, is shown in Figure 1D.

Two controls were run to validate this assay. The PCR-based method was used to calculate the ratio of array lengths from samples with predefined mixtures of cells (Supplementary Figures S4A–C). Second, cells from an expansion experiment were plated out to single cells at the end of either a 1-day or 5-day time course. PCRs and gel electrophoresis revealed the ratio of array lengths from 198 individually screened colonies closely matched the ratio of array lengths measured from the original mixed population (Supplementary Figure S4D). Newly acquired spacers from some of these individual colonies were sequenced, showing new spacers derived from either the genome or the pUC19 plasmid, depending on the strain (Supplementary Table S5).

### A model of array expansion

**Figure 2:**
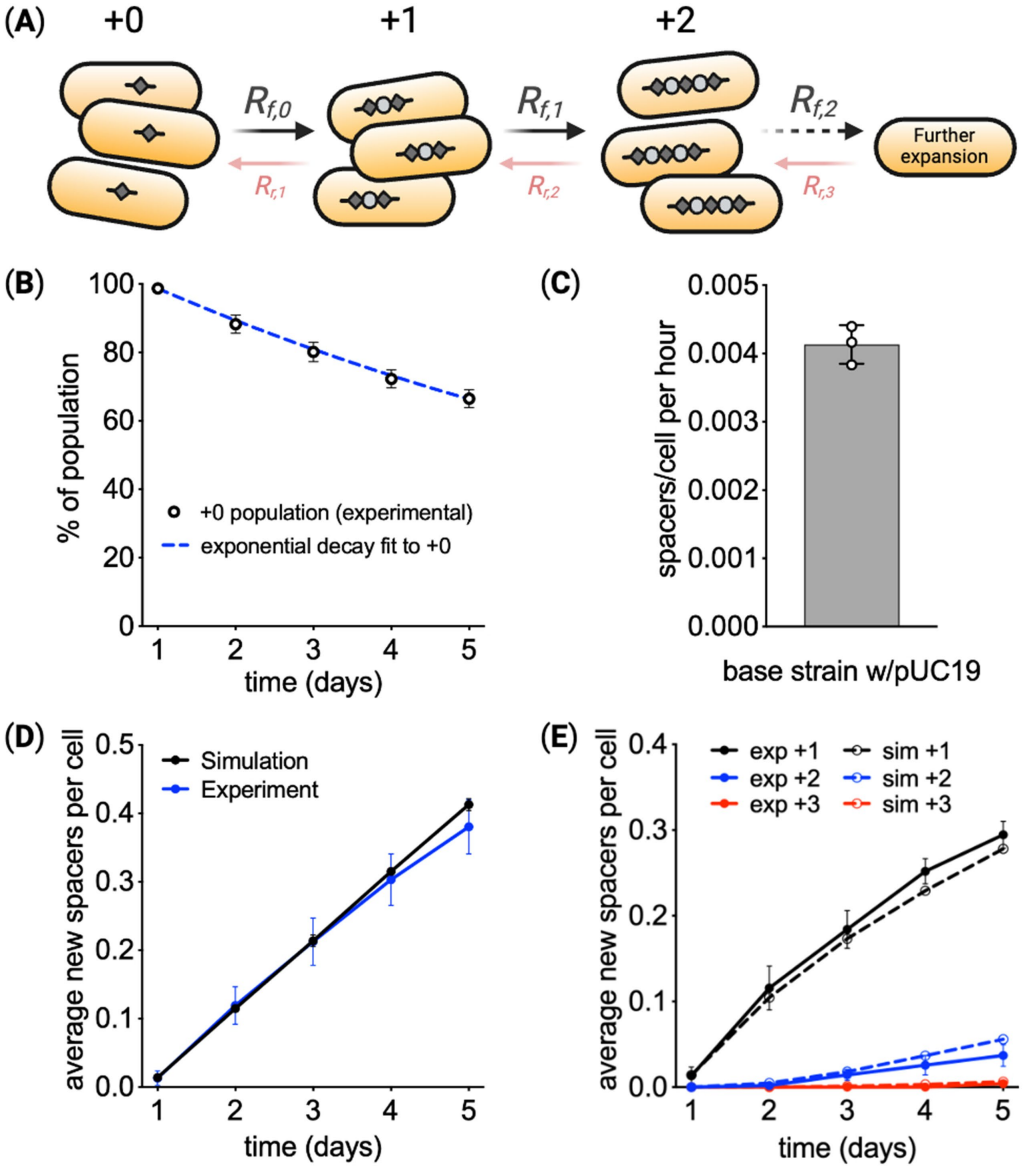
Modeling CRISPR spacer acquisition. **(A)** In the model, arrays with length *i* expand at rate *R_f, i_*, resulting in the addition of one new spacer to the array. Arrays can also lose spacers at rate *R_r, i_*. **(B,C)** Assuming cells at all array lengths have the same array expansion rate and cellular growth rate, the loss of the cells at the original array length +0 was used to calculate the rate of array expansion. **(D)** Using the calculated array expansion rate, a simulation predicted the average length of the array over time, showing deviation from experimental results at later times. **(E)** Simulation results for the percentage of cells at each array length also indicate systematic deviation from experimental results. Means are reported from three biological replicates ± SD.

Array expansion occurs as individual CRISPR arrays gain spacers in a sequential process. This process can be modeled as shown in Figure 2A. A cell with array length +0, the array length at the start of the experiment, transitions to array length +1 at a rate proportional to $R_{f}{,}_{0}$. Cells with arrays of length +1 can then transition to arrays of length +2 at a rate proportional to $R_{f}{,}_{1}$ and so on. Similarly, the model allows for contraction of the CRISPR array (spacer deletion), such that cells with an array length of +1 can transition to length +0 at rate $R_{r}{,}_{1}$. In this model, the number of cells with array length $L_{i}$ is $N_{i}$. Cells at each array length divide with growth rate constant μ. Combining these processes, the change in number of cells with array of length $L_{i}$ follows:


(3) $$\frac{dN_{i}}{dt}=N_{i}\mu -N_{i}R_{r,i}+N_{i+1}R_{r,i+1}-N_{i}R_{f,i}+N_{i-1}R_{f,i-1}\text{.}$$

and for cells with array length of +0,


(4) $$\frac{dN_{0}}{dt}=N_{0}\mu -N_{0}R_{f,0}+N_{1}R_{r,1},$$

Using these equations, the experimental data can be fit to calculate the array expansion rate. In this initial fit, the growth rate of cells and array expansion rate was assumed to be constant (i.e., does not change over time and is independent of array length).

The rate of array contraction was set to zero as no cells with contracted arrays were detected over 120 h of measurements. Contraction was monitored in two ways. First, the back end of the CRISPR array, from parental spacer-5 through the end of the array beyond parental spacer-13 was measured across a 5-day time course for the base recording strain with and without pUC19 (Supplementary Figure S8A). The standard acquisition measurements probe only the leader proximal end of the array; however, contraction of the array may occur at any point from the leader-proximal to the leader-distal end. No spacer loss was detected in the back end of the array over 5 days. Second, the leader-proximal end of the array was measured for several expanded clones with different array lengths between +1 and +5. These clones were isolated from a culture of the pUC19 recording strain that was previously induced for Cas1–Cas2 expression. A subsequent 5-day non-induction time course was run for mixtures of these expanded clones with none producing PCR bands below the starting amplicon size, indicating no appreciable loss of the newly acquired spacers (Supplementary Figure S8B). Although array contraction has been identified both experimentally and through comparative genomics (Deecker and Ensminger, 2020; Garrett, 2021), these two experiments demonstrate that spacer deletion events are insignificant in these strains over the timescale analyzed. The replacement of spacers in the array with new sequences may be possible, but was not observed in any sequenced arrays and seems unlikely to noticeably bias measurements of array dynamics.

With these assumptions, the model used the decay of the percentage of cells at the starting array length to calculate the array expansion rate. Figure 2B shows the change in the fraction of cells at the original array length over time. As derived in the Supplementary material Text S1, when assuming array expansion and cell growth are constant for all array lengths, the change in the fraction of cells at the original length follows:


(5) $$f_{0}\left(t\right)=e^{-R_{f}t}$$

Figure 2C reports the array expansion rate fitting the data from Figure 2B using Equation 5. To check if this expansion rate was consistent with the change in all array lengths over time, we simulated the fractions of cells at all array lengths over the experimental timeframe using the rate from Figure 2C. The simulation used an Euler forward algorithm and Equations 3, 4 to predict the change in array lengths within the population over time. Simulation results are shown next to experimental measurements in Figure 2D, indicating experimental measurements of average array length begin to deviate from model predictions toward the end of the experiment. Figure 2E further shows predictions of expansion at each array length systematically deviates from experimental results. These comparisons suggest some form of feedback that reduces the fraction of cells at longer array lengths.

**Figure 3:**
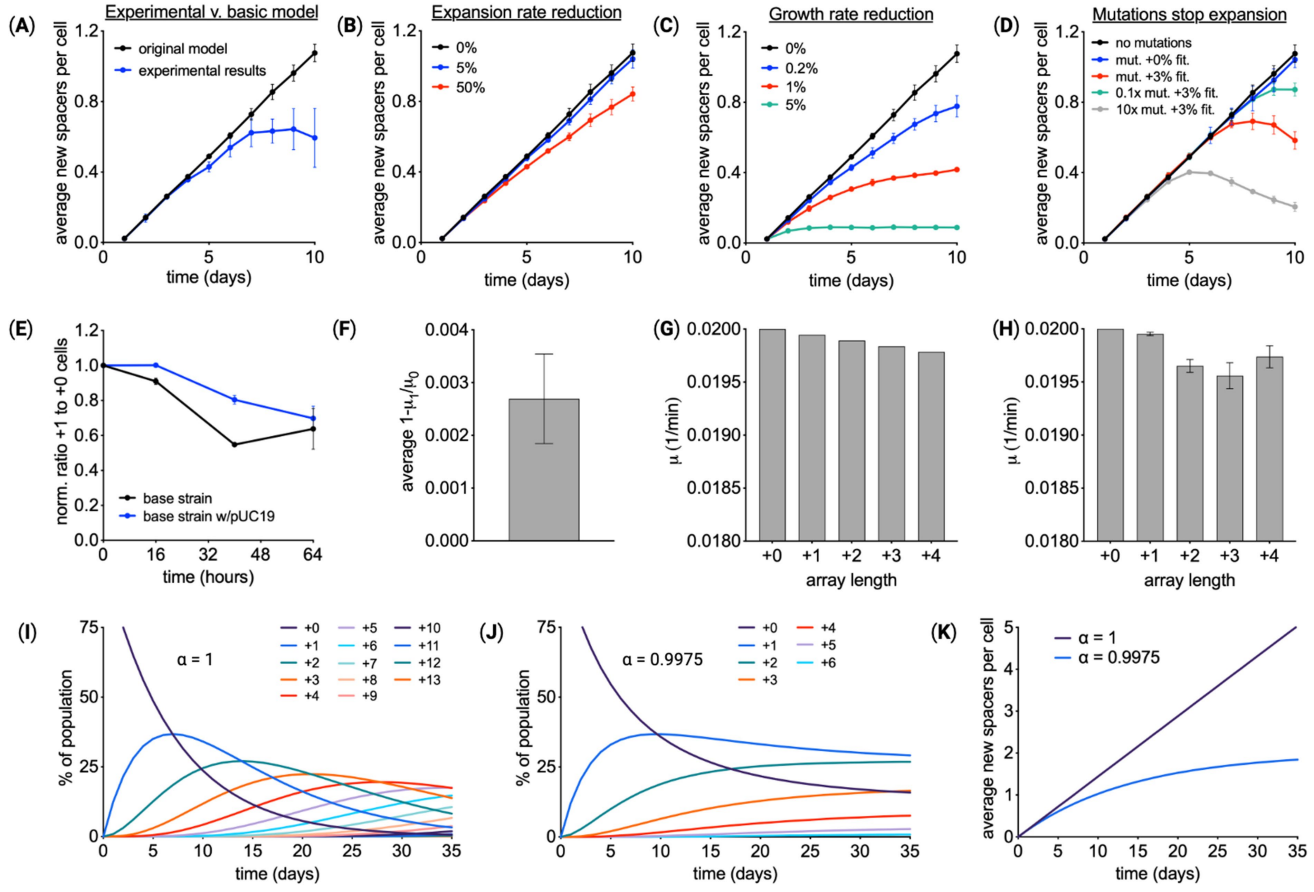
Array expansion associated with a fitness cost. **(A)** Spacer acquisition over 10 days of Cas1–Cas2 induction for the base recording strain containing pUC19. Comparing experimental results with a model that assumes the rate of array expansion and cell growth is independent of array length. Given the reduced expansion observed in experiments, modifications of the model were explored. **(B)** A model in which the array expansion decreases by X% for every new spacer. **(C)** A model in which cell growth rate slows by X% for every new spacer. **(D)** A model in which cells within the population mutate. Mutant cells have an array expansion rate of zero and may have a gain in fitness. **(E)** Experimental validation of a fitness cost associated with array expansion. Cas1–Cas2 was expressed in cells with a starting array length of +0 for 24 h, resulting in a mixed population of cells with array lengths of +0 and +1. Cells were transferred to media without inducer. Starting at 32 h the ratio of +1 and +0 cells was monitored over time using the PCR-based method. *n* = 3 for each condition. **(F)** From the change in the ratio of +1 to +0 cells, the ratio of cell growth rate, *μ*1/*μ*0 or *α_μ_*, was calculated. **(G)** This value of *α_μ_* predicts the growth rates for cells with different array lengths. **(H)** Experimental data shown in panel **(A)** was fit to calculate the growth rate for cells at each array length, revealing a similar trend of a small decrease in the growth rate as the array expanded. **(I)** Simulated spacer acquisition over 35 days with a constant acquisition rate and no fitness effects associated with array length (*α_μ_* = 0). **(J)** Simulating spacer acquisition over 35 days with a constant acquisition rate and a growth reduction of *α_μ_* = 0.9975. **(K)** The average array length over time using the simulation results from **(I)** and **(J)**. **(A–D)** means of three independent simulations ± SD. **(E–F)** show means ± SD.

To further explore the differences between model predictions and experimental data, the array expansion measurements were run to 10 days. As shown in Figure 3A, the average array length in the population increases at a lower rate over time, not following the linear growth trajectory predicted by the model. The model was adapted to include feedback related to array length. Three options for reducing the percentage of cells with longer arrays were considered. The first model variant made array expansion dependent on array length (Figure 3B), specifically,


(6) $$R_{f,i}=R_{f,0}\;\alpha_{R}^{i},$$

where $\alpha_{R}^{i}$ is a factor that reduces the rate constant for array expansion rate raised to the power i. The second model variant reduces the cell growth rate for every new spacer added to the array (Figure 3C). In this model,


(7) $$\mu_{i}=\mu_{0}\;\alpha_{\mu }^{i},$$

where $\alpha_{\mu }^{i}$ is a factor that reduces the rate constant for cell growth rate raised to the power i. The third model variant assumes that mutations appear within the population over time that deactivate spacer acquisition (Figure 3D). In simulations, the mutations occur at a frequency of 10^−6^ mutations/(cell min). 10× and 0.1× mutation frequency is 10^−5^ 1/(cell min) and 10^−7^ 1/(cell min), respectively. Mutated cells have array expansion rate constants equal to zero. The potential of mutants having a fitness advantage was also explored. These three models were compared by running simulations that approximate the experimental procedure. In the simulation, cells grow over time, and at each timepoint, individual cells add a single new spacer with probability 0.0049 1/h. When the culture reaches 10^8^ cells, a small fraction of cells, 0.01%, are inoculated into fresh media. Transferred cells are chosen randomly from the population.

Comparison of the modeling results points to a fitness cost to array expansion, see also Supplementary Figure S9. The reduction in the array expansion rate would be very large to account for the trend in average array length over time, more than a 50% reduction in the expansion rate for every new spacer. The mutations that cease array expansion would have to be frequent and have a fitness benefit of several percent. Conservatively, the rate of mutations that would impact spacer acquisition would be less than 10^−8^ 1/(cell min) (Foster et al., 2015), so the simulations represent the extreme case of an abnormal frequency of mutation. Conversely, the reduction in fitness that results in the average array length leveling off to 0.5 spacer per cell would be between 0.2 and 1%, only a small penalty.

Testing the fitness hypothesis experimentally, cells with arrays of different lengths were competed over time. First, a clonal culture with a starting array length of +0 was expanded for 24 h, via Cas1–Cas2 induction. This resulted in a culture containing a mixture of cells with mostly +0 and +1 array lengths. Cells were then transferred to media without inducer, and after waiting 8 h for expansion to cease, the ratio of +1 to +0 cells was monitored via the PCR-based method over 64 h (Figure 3E and Supplementary Figure S5). This experiment was performed using the base recording strain with and without pUC19. Results showed +0 cells outcompeted +1 cells over time. From these measurements, the cells with the longer array had a growth rate constant ~0.27% smaller (Figure 3F). Growth rates for cells with different array lengths were predicted using this measured deficit (Figure 3G). To see whether these predicted growth rates matched experiments, the experimental data from Figure 3A were refit, with the growth rate of cells at each array length as free parameters. The extracted growth rates were similar to predictions based on the competition experiment (Figure 3H). Additional competition experiments between individual strains with array length +1 and the unexpanded strain revealed variable fitness consequences for array expansion that was on average slightly negative (Supplementary Figure S5E).

To explore the consequences of this fitness change, spacer acquisition was simulated over 35 days. Array expansion in populations without a fitness reduction (Figure 3I) was compared to populations with a fitness reduction (Figure 3J). Both models use the same rate of spacer acquisition, highlighting the impact of fitness on average array length over time (Figure 3K).

### Cellular parameters modulate spacer acquisition rates

**Figure 4:**
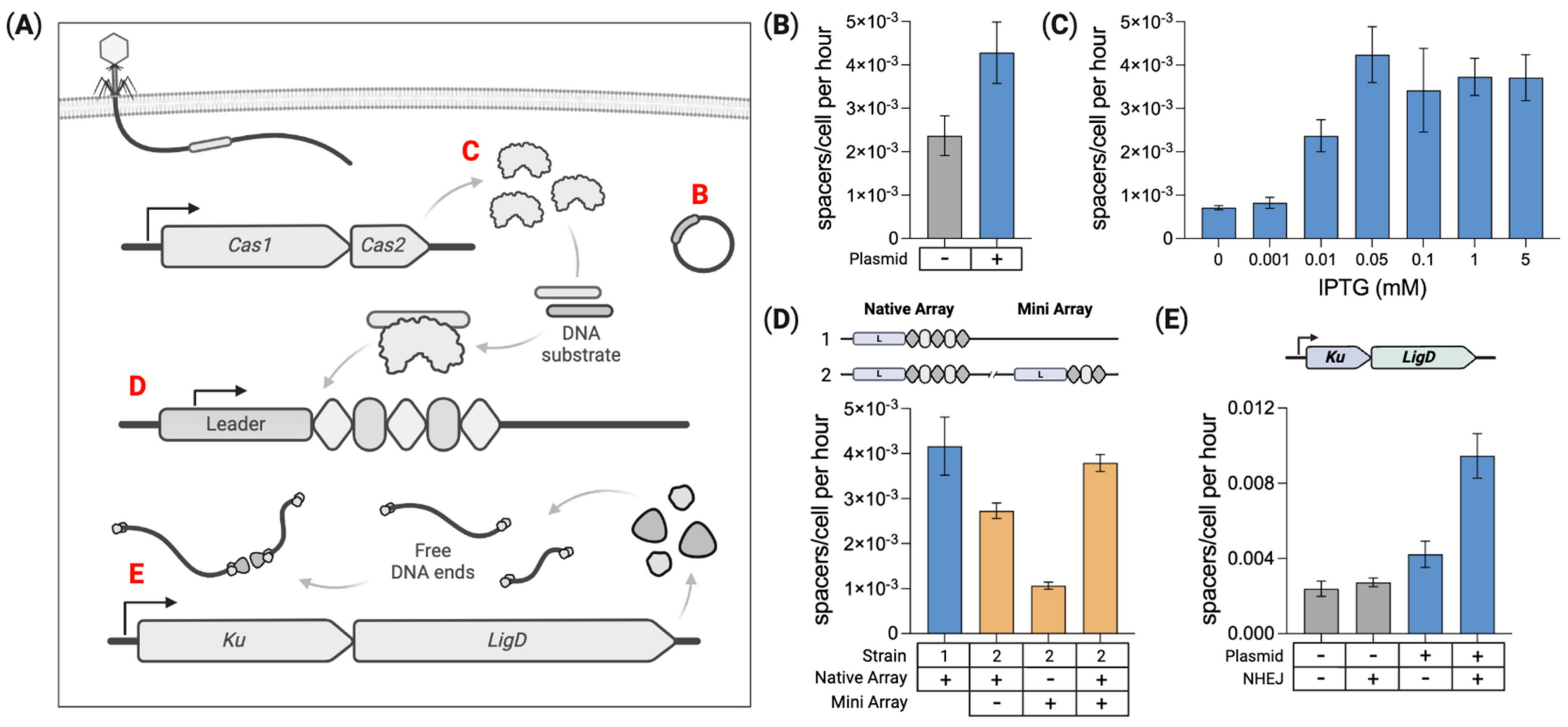
Intracellular parameters modulate spacer acquisition. **(A)** Several factors are identified that modulate spacer acquisition: Mobile genetic elements (e.g., plasmids), Cas1–Cas2 expression level, number of CRISPR arrays, and Ku–LigD expression listed as **(B–E)**, respectively. **(B)** Quantifying spacer acquisition rates from Cas1–Cas2 induction time course experiments. Acquisition rates are shown for the base spacer recording strain with and without the pUC19 plasmid. **(C)** Modulating Cas1–Cas2 expression by varying the IPTG dose and measuring the corresponding spacer acquisition rates for the base recording strain containing pUC19. **(D)** Quantifying spacer acquisition rates in the base recording strain containing pUC19 with either one or two chromosome-based CRISPR arrays. **(E)** Impact of DNA end-joining machinery (Ku + LigD) on spacer acquisition rates in the base strain with and without pUC19. For all charts here, means of three biological replicates ± SD are reported. Spacer acquisition rates were generated by fitting to constant acquisition rate and array-length-dependent fitness reduction represented by *α_μ_*. Statistical comparisons are provided in Supplementary Table 4.

Then, we examined how the rate of CRISPR array expansion is affected by cellular parameters. As shown in Figure 4A, array expansion involves Cas1–Cas2 DNA protospacer substrate processing prior to insertion as a new spacer into the array. We hypothesized parameters that influence spacer acquisition rates include the expression level of Cas1 and Cas2 proteins, the availability of DNA substrate, and the number of CRISPR arrays within the cell. Array expansion rates were measured in 5-day Cas1–Cas2 induction experiments that modulated these three parameters. Rates were generated by fitting the expansion data for the array expansion rate, from Equation 6, or the fitness penalty $\alpha_{\mu }$ in Equation 7. All $\alpha_{\mu }$ values from these experiments can be found in Supplementary Figure S10.

### DNA substrate

Our base spacer recording strain does not contain any plasmids, acquiring exclusively self-genome-derived spacers (Supplementary Table S1). When present, intracellular mobile genetic elements (MGEs) such as plasmids and bacteriophage contribute to the Cas1–Cas2 substrate pool (Sheth et al., 2017; Levy et al., 2015). We measured spacer acquisition in the base strain with and without the high copy number plasmid pUC19. With no plasmid, the acquisition rate was 2.37E−3 spacers/cell per hour. The rate increased roughly 1.8× to 4.28E−3 spacers/cell per hour with pUC19 present, Figure 4B. A few dozen colonies were PCR screened from these cultures at the end of the experiment to identify clones with an expanded array (Supplementary Table S5). From the plasmid-free strain, 23 new spacers were sequenced with all matching sequences from the host chromosome. For the strain containing pUC19, 18 new spacers were sequenced and identified, with 3 derived from pUC19 and 15 from the host chromosome. Shown previously, the presence of a high copy number plasmid can significantly increase the integration of host genome-derived spacers (Sheth et al., 2017).

### Cas1–Cas2 expression

The genome-integrated Cas1–Cas2 operon is controlled by a T7-lac inducible promoter expressed when cells are dosed with both arabinose and IPTG (Yosef et al., 2012). IPTG releases the repressor from an operator upstream of Cas1–Cas2, and arabinose induces the expression of genomic T7 RNA-polymerase required for Cas1–Cas2 transcription. We sought to induce a range of Cas1–Cas2 expression levels and measure the corresponding spacer acquisition rates. This was done by titrating the IPTG dose with a fixed arabinose concentration (0.2%) and measuring spacer acquisition over 5 days in the base strain containing pUC19. A control condition with no arabinose or IPTG was run, revealing no detectable spacer acquisition over 5 days (Supplementary Figures S7A,B), indicating strong repression in the absence of both inducers. Seven arabinose-dosed conditions were examined with the IPTG dose ranging between 0 and 5 mM. The 0 mM IPTG condition produced a low, but detectable level of array expansion, indicating slightly leaky expression with arabinose alone, Figure 4C. The culture dosed with 0.05 mM IPTG produced the fastest rates of spacer acquisition. This experiment was also performed in the plasmid-free base strain, showing a similar trend in spacer acquisition rates for the corresponding IPTG doses (Supplementary Figure S11).

### CRISPR array copy number

To determine whether multiple arrays affect spacer acquisition rates per cell, we compared acquisition in strains containing one or two CRISPR arrays within the host chromosome. The second array was derived from the native *E. coli* CRISPR locus and is hereafter referred to as the “mini” array as it contains just two repeats flanking a single parental spacer (Supplementary Figure S12). This mini-array was integrated ~1.8Mbp away from the native CRISPR locus. Using unique pairs of primers, the expansion of each array was independently monitored through a 5-day time course. The pUC19 plasmid was added to both strains to enhance spacer acquisition rates. 0.05 mM IPTG was used for Cas1–Cas2 induction.

As shown in Figure 4D, both arrays in the two-array strain expanded slower than the single acquisition locus of the one-array strain. However, the average number of spacers acquired per cell for the two arrays combined was about the same as spacer acquisition in the one-array strain. This suggests the acquisition of new spacers was roughly split between the two arrays without changing the overall rate of spacer acquisition per cell. In the two-array strain, the added mini-array expanded more slowly than the native array, potentially due to the native array being closer to the chromosomal origin of replication (oriC), and therefore having a higher average copy number than the mini-array (Skovgaard et al., 2011). Replicating bacterial cultures contain greater sequence copy numbers for sequences closer to the oriC. This copy number gradient relative to the oriC has been shown in genomic DNA extracted from *E. coli* BL21-AI (Levy et al., 2015).

### Expression of heterologous DNA end-joining genes

We hypothesized the Cas1–Cas2 DNA substrate pool may be impacted by pathways that can protect and join together free DNA ends in the cell. We tested this hypothesis by introducing a bacterial non-homologous end-joining (NHEJ) system made up of two genes that specifically serve this purpose. *Escherichia coli* does not have a native NHEJ pathway, but heterologous NHEJ can be introduced. This simple, two-component NHEJ system found in some bacterial species utilizes the genes Ku and LigD for non-homologous end joining (Shuman and Glickman, 2007). Ku binds to free DNA ends protecting them from exonuclease degradation and LigD ligates these DNA ends together (Aniukwu et al., 2008). We reasoned that these functions may preserve more Cas1–Cas2 substrate by protecting intracellular DNA debris. Ku and LigD native to *Mycobacterium smegmatis* were assembled into an operon and genome integrated into our base recording strain. Spacer acquisition rates were measured for this strain with and without pUC19. Figure 4E shows that expression of Ku and LigD increased the spacer acquisition rate by ~14% for the base recording strain and ~124% in the strain containing pUC19. Gel image comparisons for the pUC19 strains can be found in Supplementary Figure S13.

### Enhanced array expansion confers increased phage protection

To test whether array expansion rate influences phage resistance, CRISPR interference-enabled spacer acquisition strains with different array expansion rates to be infected with bacteriophage Lambda. We utilized an infection protocol that tracks cell density post-phage inoculation (Rajnovic et al., 2019). OD600 measurements tracked culture growth in response to an infection. OD600 curves were compared for infected and uninfected cultures to determine the extent of phage-induced growth inhibition.

**Figure 5:**
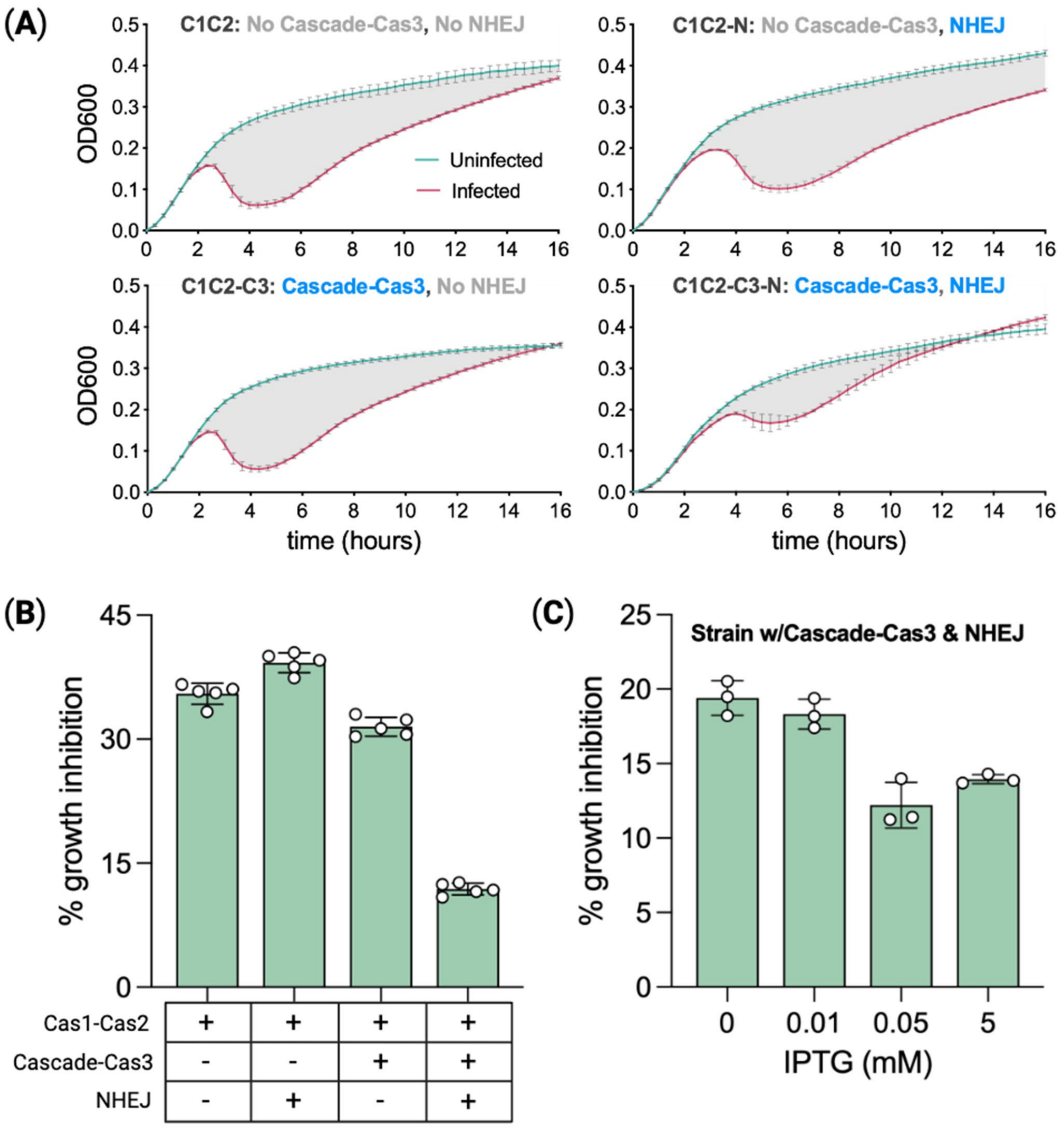
Phage protection is correlated with spacer acquisition rates. **(A)** Spacer recording strains with or without CRISPR effector machinery (Cascade–Cas3), and with or without heterologous NHEJ (Ku–LigD), were infected with bacteriophage Lambda to measure the degree of growth inhibition relative to uninfected controls. OD600 measurements tracked culture growth over 16 h. Means of five biological replicates ± SD are reported. **(B)** Percent growth inhibition from **(A)** calculated as the percent difference in area under the curve for infected versus uninfected. The period assessed was the 15 h from when cultures reached a growth rate of 0.001 OD units per minute. Expression (+) or not (−) for relevant genes is indicated below the chart. **(C)** Percent growth inhibition for the most protected strain (effector + NHEJ competent) treated with a range of IPTG doses to modulate Cas1–Cas2 expression. Means of three biological replicates ± SD are reported. Statistical comparisons are provided in Supplementary Table 4.

All strains used in this infection assay were plasmid-free, capable of acquiring spacers derived from either the infecting bacteriophage or the self-genome. Four strains were run in this assay to compare their relative resistance to phage infection (Figure 5A), C1C2: the base acquisition strain containing only Cas1–Cas2, C1C2-N: the same base strain with the addition of NHEJ, C1C2-C3: the base strain with all CRISPR machinery but not NHEJ, and C1C2-C3-N: the base strain with all CRISPR machinery and NHEJ. Cultures were inoculated with or without Lambda phage and dosed with spectinomycin (50 μg/mL), IPTG (0.05 mM), arabinose (0.2%), and rhamnose (0.1%). These cultures were distributed across a 96-well plate and run on a plate reader for 21 h with OD600 data collected every 20 min.

For each strain, we calculated the area under the curve for infected and uninfected cultures to quantify phage-induced growth inhibition. The difference in the respective areas is the percent growth inhibition relative to uninfected cultures (Rajnovic et al., 2019). For each growth curve, the area was calculated from a start point of detection (SPD) to an endpoint of detection (EPD). SPD is defined as the threshold at which the culture growth rate reaches 0.001 OD units per minute, and the EPD is 15 h post-SPD. The base recording strain C1C2, lacking CRISPR interference and NHEJ machinery, had a phage-induced growth inhibition of ~35%, Figure 5B. C1C2-N growth was inhibited by ~39%. C1C2-C3, capable of utilizing spacer-derived crRNAs for targeting, had growth inhibited by ~31% and the strain combining the full CRISPR system with NHEJ (C1C2-C3-N) had a 3-fold reduction in growth inhibition at ~12%.

A second experiment using just C1C2-C3-N was run to directly test the hypothesis that phage protection varies with spacer acquisition rates. The IPTG dose range experiment in the base recording strain showed that spacer acquisition rates are modulated with Cas1–Cas2 expression levels. In this experiment, we inoculated C1C2-C3-N with one of four IPTG doses from 0 mM to 5 mM while keeping the arabinose dose fixed (0.2%) and also inducing Cascade–Cas3 expression with rhamnose (0.1%) as seen in Figure 5C. The experiment was run in the same way as the previous plate-reader time course. Phage-induced growth inhibition was lowest (~12%) for the IPTG dose previously shown to produce the highest spacer acquisition rate (0.05 mM), as seen in Figure 4C, whereas inhibition in the 0 mM IPTG cultures was ~60% higher. Spacer acquisition rates associated with Cas1–Cas2 expression levels corresponded in rank order to the degree of protection from phage infection, indicating a positive correlation between spacer acquisition rate and phage protection. Approximately 20% growth inhibition for cells expressing NHEJ at 0 IPTG as compared to 30% growth inhibition for cells without NHEJ at 0.05 mM IPTG suggests the potential for NHEJ to increase spacer acquisition is exaggerated in phage infection cells. A similar increase in the expansion rate for NHEJ-expressing cells with plasmid compared to cells without plasmid was observed in Figure 4E.

From the most-protected C1C2-C3-N strain, 14 expanded-array clones isolated post-infection were sequenced across the CRISPR array to identify the newly acquired spacer sequences. Interestingly, all 15 of the spacers identified were derived from the host genome. These 15 spacer sequences are listed in Supplementary Table S5.

## Discussion

In this study, we used a synthetic CRISPR/Cas system derived from *E. coli* to characterize rates of spacer acquisition in the absence of interference machinery. Establishing a baseline spacer acquisition rate for this system allowed us to identify intracellular factors that modulate array expansion rates. We identified three intracellular factors that affect these rates: (1) Cas1–Cas2 protospacer substrate, (2) Cas1–Cas2 expression levels, and (3) the number of arrays within the genome. Introducing a high copy number plasmid (pUC19) into our base recording strain increases the Cas1–Cas2 protospacer substrate concentration (Levy et al., 2015; Sheth et al., 2017) resulting in a nearly 2-fold increase in the rate of spacer acquisition (Figure 4B). Utilizing an inducible promoter in the Cas1–Cas2 operon, we varied the expression levels for these adaptation genes and measured a corresponding range of spacer acquisition rates (Figure 4C). The fastest rates of spacer acquisition occurred at a midrange IPTG concentration. With Cas1 and Cas2 proteins coalescing to form a 6-subunit integrase complex (Cas1)_2_(Cas2)_2_(Cas1)_2_, saturating expression (≥1 mM IPTG) may produce larger, non-functional protein aggregates, reducing spacer acquisition potential. With a second array introduced into the base strain, the rate of new spacers acquired per cell did not change much as the acquisition rate for each array was roughly half that of the single-array strain (Figure 4D). We hypothesized that expression of heterologous NHEJ genes Ku and LigD may enhance spacer acquisition by stabilizing DNA fragments, increasing the concentration of Cas1–Cas2 substrate within cells. Ku binds to and protects DNA ends from exonucleolytic degradation and LigD can ligate these ends together. We discovered that NHEJ expression does increase spacer acquisition, with rates boosted as much as ~124% relative to the non-NHEJ control (Figure 4E). This *Mycobacterium smegmatis*-derived NHEJ construct was also introduced into a strain with a fully functional Type I-E CRISPR system and found to provide a 3-fold increase in protection from phage infection. These strategies to control spacer acquisition may help to better understand and engineer CRISPR/Cas systems in bacteria (Shivram et al., 2021). Prior study with Type II-A CRISPR showed that transcription of the CRIPSR array may affect spacer acquisition rates. Array transcription is a mechanism to resolve the post-synaptic complex to complete spacer integration (Budhathoki et al., 2020). It is not clear whether array transcription would also influence spacer acquisition for Type I-E CRISPR. In this study, array transcription was not modified or intentionally regulated; therefore, our measurements would not reveal any impacts of array transcription.

Using the data from these studies, we modeled naïve spacer acquisition in this system. The basic model, considering constant spacer acquisition and cell growth, predicted a linear increase in average array length per cell over time, which was not supported by experimental data (Figure 2D). Several other variables were modeled including slowed acquisition, fitness effects associated with expanded populations, and array-expansion inactivation (mutation). Both the modeling (Figure 3C and Supplementary Figure S9) and experimental data (Figure 3E) suggest fitness effects linked to array-expanded populations are the source of this disparity. Although CRISPR/Cas self-targeting of host RNA has previously been identified as an infection defense strategy, a mechanism for spacers influencing host fitness in the absence of interference machinery has not been detected (Meeske et al., 2019). We suspect that precursor crRNA transcripts in cells lacking Cascade-mediated processing may interact with complementary sequences in the genome or within plasmids. Genome-complementary RNA sequences may impact host gene expression, producing net fitness effects. It is not known whether antisense sequences within unprocessed CRISPR-array transcripts can affect the translation of mRNAs, but bacterial RNA interference (RNAi) mechanisms are known to be involved in post-transcriptional gene silencing (PTGS) (Lioliou et al., 2010; Rusk, 2012; Saberi et al., 2016). Alternatively, it has been shown that overexpression of Cas1–Cas2 can result in non-canonical spacer integrations into non-array regions within the genome, potentially resulting in fitness effects (Nivala et al., 2018). As shown in Supplementary Figure S5, the fitness impact of array expansion was variable, presumably depending upon the sequence of the acquired spacer. It is intriguing to consider whether a gene regulatory effect from CRISPR-array transcripts, in the absence of additional interference machinery, could be a secondary and more primitive function of a CRISPR array.

Several emerging technologies utilize spacer acquisition as a tool for various applications including recording the occurrence and order of events in cellular environments resulting in transcription (Munck et al., 2020; Sheth and Wang, 2018; Lear and Shipman, 2023) and converting digital data to biological storage in CRISPR arrays (Shipman et al., 2017; Yim et al., 2021). A broader understanding of spacer acquisition and the factors affecting rates of spacer uptake may enable tuning the frequency of these events to optimal rates for specific applications. In addition, engineering conditions to maximize spacer acquisition rates may increase the probability of recording rare events.

We show that faster CRISPR adaptation provides greater protection from phage infection in our engineered *E. coli* host with constant CRISPR/Cas induction (Figure 5A). As host cells acquire resistance to infection, the phage can coevolve through escape mutations (Barrangou et al., 2007), with host spacer acquisition (naïve and primed), a key rate-limiting factor for the adaptive immune response (Sternberg et al., 2016; Heler et al., 2017; Datsenko et al., 2012; Staals et al., 2016). In this sense, faster spacer acquisition would increase survival during infection. However, the benefit/cost ratio of spacer integration rates may be proportional to the threat of the infectious agent as potentially damaging self-targeting spacers can also be acquired. Strains with very long CRISPR arrays either have an increased benefit from a greater repertoire of spacer sequences or somehow have managed to reduce the costs associated with array growth. Over much longer times, the rate of array contraction should play a role in setting the size of the array. Although a bias exists for the acquisition of spacers derived from MGEs, genome-derived spacers are also acquired during infection (Shipman et al., 2016; Levy et al., 2015). Bacteria are generally deficient in robust double-strand DNA break repair pathways (Finger-Bou et al., 2020; Wimmer and Beisel, 2019), reducing the probability of self-targeting survival. Wild-type bacterial cells generally maintain strong regulatory control over CRISPR/Cas to maximize the benefit of expression in dynamic natural environments (Markulin, 2020; Patterson et al., 2016). Increasing spacer acquisition rates to enhance CRISPR/Cas efficiency may help improve bacteriophage resistance needed in fermentation and other industrial processes (Deem, 2020; Garneau and Moineau, 2011; Maguin et al., 2022), with consideration for temporal control of expression. The fact that no phage-derived spacers were detected post-infection is puzzling, although prior study has shown that CRISPR-associated defense against phages does not always lead to the maintenance of phage-targeting spacers (Strotskaya et al., 2017).

Ku and LigD NHEJ genes function naturally to repair double-strand DNA breaks. In the context of CRISPR spacer acquisition, however, we hypothesized a role for these genes in producing a larger Cas1–Cas2 DNA substrate pool. We verified increased acquisition rates in strains expressing these NHEJ genes. This result implies that other pathways may also impact spacer acquisition rates by altering the concentration of intracellular DNA debris. In *E. coli*, exonucleases RecBCD and SbcCD degrade DNA from free ends. Directly regulating the expression of these genes or expressing an exonuclease inhibitor such as Gam may also result in enhanced spacer acquisition rates. Discovery of other native and heterologous factors affecting CRISPR efficiency, as well as engineering and evolving improvements, may further expand application potentials for both CRISPR adaptation alone and for functional CRISPR/Cas defense.

## Data Availability

The original contributions presented in the study are included in the article/Supplementary material, further inquiries can be directed to the corresponding author.
