# Supplementary material for: Assessing spacer acquisition rates in E. coli type I-E CRISPR arrays
Source: Front Microbiol. 2025 Jan 20;15:1498959. doi: 10.3389/fmicb.2024.1498959 (PMC11788318; doi:10.3389/fmicb.2024.1498959)
Supplement: Supplementary file 2 [file Data_Sheet_1.PDF]

```
//  Supplemental code 3
//  main.cpp
//  cell growth simulation
//
//  Created by 古德猫宁 Harry on 5/21/24.
//

#include <iostream>
#include <vector>
#include <cmath>
//#include <gnuplot-iostream.h>
using namespace std;


void one_day_growth(int date, double growth_rate, double expansion_fraction, double
fitness_factor, double contraction_rate, double acquisition_rate, double mutation,
double red_expansion, vector<double>& plus_0, vector<double>& plus_1,
vector<double>& plus_2, vector<double>& plus_3, vector<double>& plus_4,
vector<double>& plus_5, vector<double>& plus_0_mut, vector<double>& plus_1_mut,
vector<double>& plus_2_mut, vector<double>& plus_3_mut, vector<double>& plus_4_mut,
vector<double>& plus_5_mut, double dt);

int main(int argc, const char * argv[]) {
    double expansion_fraction = 0.9; //fraction of expansion rate for higher array
length (1)
    double fitness_factor = 1; //fitness of expanded clones compared to unexpanded
(1)
    double contraction_rate = 0; //contraction throughout whole culture (0)
    double red_expansion = 1; //fraction array expansion rate over time (1)

    double time_24[4] = {93.43438201, 6.56561799, 0, 0};
    int cell_count_init = 50000; //number of cells to start with (do not change)
    double growth_rate = 0.02; //growth rate per minute (do not change)
    double acquisition_rate = 0.00686/60; //array expansion per minute
    double mutation = 0/1440; //rate of mutation (0)

    double dt = 0.01; //minute per loop (60 = 1 hour)
    int days = 5;

    vector<double> plus_0;
    plus_0.push_back(cell_count_init*time_24[0]);
    vector<double> plus_0_mut;
    plus_0_mut.push_back(0);
    vector<double> plus_1;
    plus_1.push_back(cell_count_init*time_24[1]);
    vector<double> plus_1_mut;
    plus_1_mut.push_back(0);
    vector<double> plus_2;
    plus_2.push_back(cell_count_init*time_24[2]);
    vector<double> plus_2_mut;
```

```
    plus_2_mut.push_back(0);
    vector<double> plus_3;
    plus_3.push_back(cell_count_init*time_24[3]);
    vector<double> plus_3_mut;
    plus_3_mut.push_back(0);
    vector<double> plus_4;
    plus_4.push_back(0);
    vector<double> plus_4_mut;
    plus_4_mut.push_back(0);
    vector<double> plus_5;
    plus_5.push_back(0);
    vector<double> plus_5_mut;
    plus_5_mut.push_back(0);

    for(int i = 0; i < days; i++){ //i is number of days
        one_day_growth(i, growth_rate, expansion_fraction, fitness_factor,
contraction_rate, acquisition_rate, mutation, red_expansion, plus_0, plus_1, plus_2,
plus_3, plus_4, plus_5, plus_0_mut, plus_1_mut, plus_2_mut, plus_3_mut, plus_4_mut,
plus_5_mut, dt);

        double total_cell =
plus_0.back()+plus_1.back()+plus_2.back()+plus_3.back()+plus_4.back()+plus_5.back()+
plus_0_mut.back()+plus_1_mut.back()+plus_2_mut.back()+plus_3_mut.back()+plus_4_mut.b
ack()+plus_5_mut.back();
        double innoculate_fraction = 50000/total_cell;

        //cout << "innoculate_fraction" << innoculate_fraction << endl;

        plus_0.back() *= innoculate_fraction;
        plus_0_mut.back() *= innoculate_fraction;
        plus_1.back() *= innoculate_fraction;
        plus_1_mut.back() *= innoculate_fraction;
        plus_2.back() *= innoculate_fraction;
        plus_2_mut.back() *= innoculate_fraction;
        plus_3.back() *= innoculate_fraction;
        plus_3_mut.back() *= innoculate_fraction;
        plus_4.back() *= innoculate_fraction;
        plus_4_mut.back() *= innoculate_fraction;
        plus_5.back() *= innoculate_fraction;
        plus_5_mut.back() *= innoculate_fraction;
    }

    vector<double> fraction_p0;
    vector<double> fraction_p1;
    vector<double> fraction_p2;
    vector<double> fraction_p3;
    vector<double> fraction_p4;
    vector<double> fraction_p5;

    for(int i = 0; i < 4*1440/dt; i++){
```

```
        double tot_cell =
plus_0[i]+plus_1[i]+plus_2[i]+plus_3[i]+plus_4[i]+plus_5[i]+plus_0_mut[i]+plus_1_mut
[i]+plus_2_mut[i]+plus_3_mut[i]+plus_4_mut[i]+plus_5_mut[i];
        //cout << "total cells: " << tot_cell << endl;
        fraction_p0.push_back((plus_0[i]+plus_0_mut[i])/tot_cell*100);
        fraction_p1.push_back((plus_1[i]+plus_1_mut[i])/tot_cell*100);
        fraction_p2.push_back((plus_2[i]+plus_2_mut[i])/tot_cell*100);
        fraction_p3.push_back((plus_3[i]+plus_3_mut[i])/tot_cell*100);
        fraction_p4.push_back((plus_4[i]+plus_4_mut[i])/tot_cell*100);
        fraction_p5.push_back((plus_5[i]+plus_5_mut[i])/tot_cell*100);

    }
    //cout << "fraction_p0 size: " << fraction_p0.size() << endl;
    cout << "fraction_p0: " << endl;
    for(int i = 0; i < fraction_p0.size(); i++){
        if(i % int(24*60/dt) == 0){
            cout << fraction_p0[i] << endl;
        }
    }
    cout << fraction_p0.back() << endl;
    cout << "fraction_p1: " << endl;
    for(int i = 0; i < fraction_p1.size(); i++){
        if(i % int(24*60/dt) == 0){
            cout << fraction_p1[i] << endl;
        }
    }
    cout << fraction_p1.back() << endl;
    cout << "fraction_p2: " << endl;
    for(int i = 0; i < fraction_p2.size(); i++){
        if(i % int(24*60/dt) == 0){
            cout << fraction_p2[i] << endl;
        }
    }
    cout << fraction_p2.back() << endl;
    cout << "fraction_p3: " << endl;
    for(int i = 0; i < fraction_p3.size(); i++){
        if(i % int(24*60/dt) == 0){
            cout << fraction_p3[i] << endl;
        }
    }
    cout << fraction_p3.back() << endl;
    cout << "fraction_p4: " << endl;
    for(int i = 0; i < fraction_p4.size(); i++){
        if(i % int(24*60/dt) == 0){
            cout << fraction_p4[i] << endl;
        }
    }
    cout << fraction_p4.back() << endl;
    cout << "fraction_p5: " << endl;
    for(int i = 0; i < fraction_p5.size(); i++){
```

```
        if(i % int(24*60/dt) == 0){
            cout << fraction_p5[i] << endl;
        }
    }
    cout << fraction_p5.back() << endl;
}

void one_day_growth(int date, double growth_rate, double expansion_fraction, double
fitness_factor, double contraction_rate, double acquisition_rate, double mutation,
double red_expansion, vector<double>& plus_0, vector<double>& plus_1,
vector<double>& plus_2, vector<double>& plus_3, vector<double>& plus_4,
vector<double>& plus_5, vector<double>& plus_0_mut, vector<double>& plus_1_mut,
vector<double>& plus_2_mut, vector<double>& plus_3_mut, vector<double>& plus_4_mut,
vector<double>& plus_5_mut, double dt){
    //cout << cell_count_init << rep_period << expansion_fraction << fitness_factor
<< contraction_rate << endl;
    int timepoint = int(1440/dt);
//    cout << "timepoint: " << timepoint << endl;

    for(int i = 1; i <= timepoint; i++){
//        cout << "date: " << date;
//        cout << "in if: date: " << date << "i: " << i << endl;
        double a_1 = plus_0.back()*acquisition_rate*dt*pow(red_expansion, date);
//inflow to plus 1 without contraction or outflow to plus 2
        double a_2 =
plus_1.back()*acquisition_rate*expansion_fraction*dt*pow(red_expansion, date);
        double a_3 = plus_2.back()*acquisition_rate*pow(expansion_fraction,
2)*dt*pow(red_expansion, date);
        double a_4 = plus_3.back()*acquisition_rate*pow(expansion_fraction,
3)*dt*pow(red_expansion, date);
        double a_5 = plus_4.back()*acquisition_rate*pow(expansion_fraction,
4)*dt*pow(red_expansion, date);
        double a_6 = plus_5.back()*acquisition_rate*pow(expansion_fraction,
5)*dt*pow(red_expansion, date);

        double c_1 = plus_1.back()*contraction_rate*dt;
        double c_2 = plus_2.back()*contraction_rate*dt;
        double c_3 = plus_3.back()*contraction_rate*dt;
        double c_4 = plus_4.back()*contraction_rate*dt;
        double c_5 = plus_5.back()*contraction_rate*dt;

        double mut_0 = plus_0.back()*mutation*dt;
        double mut_1 = plus_1.back()*mutation*dt;
        double mut_2 = plus_2.back()*mutation*dt;
        double mut_3 = plus_3.back()*mutation*dt;
        double mut_4 = plus_4.back()*mutation*dt;
        double mut_5 = plus_5.back()*mutation*dt;

        //fitness factor start at plus 2, inflow/outflow first then grow
//
```

```
plus_0.push_back((plus_0.back()-temp_1-mut_0)*(1+growth_rate*dt-contraction_rate*dt)
);
//
plus_1.push_back((plus_1.back()+temp_1-temp_2-mut_1)*(1+growth_rate*dt-contraction_r
ate*dt));
//
plus_2.push_back((plus_2.back()+temp_2-temp_3-mut_2)*(1+growth_rate*pow(fitness_fact
or,1)*dt-contraction_rate*dt));
//
plus_3.push_back((plus_3.back()+temp_3-temp_4-mut_3)*(1+growth_rate*pow(fitness_fact
or,2)*dt-contraction_rate*dt));
//
plus_4.push_back((plus_4.back()+temp_4-temp_5-mut_4)*(1+growth_rate*pow(fitness_fact
or,3)*dt-contraction_rate*dt));
//
plus_5.push_back((plus_5.back()+temp_5-temp_6-mut_5)*(1+growth_rate*pow(fitness_fact
or,4)*dt-contraction_rate*dt));
//
//        plus_0_mut.push_back((plus_0_mut.back()+mut_0)*(1+growth_rate*dt));
//        plus_1_mut.push_back((plus_1_mut.back()+mut_1)*(1+growth_rate*dt)+mut_1);
//
plus_2_mut.push_back((plus_2_mut.back()+mut_2)*(1+growth_rate*pow(fitness_factor,1)*
dt));
//
plus_3_mut.push_back((plus_3_mut.back()+mut_3)*(1+growth_rate*pow(fitness_factor,2)*
dt));
//
plus_4_mut.push_back((plus_4_mut.back()+mut_4)*(1+growth_rate*pow(fitness_factor,3)*
dt));
//
plus_5_mut.push_back((plus_5_mut.back()+mut_5)*(1+growth_rate*pow(fitness_factor,4)*
dt));

        //fitness factor start at plus 1, grow then inflow/outflow

plus_0.push_back(plus_0.back()*(1+growth_rate*dt-contraction_rate*dt)-a_1-mut_0+c_1)
;

plus_1.push_back(plus_1.back()*(1+growth_rate*pow(fitness_factor,1)*dt)+a_1-a_2-mut_
1-c_1+c_2);

plus_2.push_back(plus_2.back()*(1+growth_rate*pow(fitness_factor,2)*dt-contraction_r
ate*dt)+a_2-a_3-mut_2-c_2+c_3);

plus_3.push_back(plus_3.back()*(1+growth_rate*pow(fitness_factor,3)*dt-contraction_r
ate*dt)+a_3-a_4-mut_3-c_3+c_4);

plus_4.push_back(plus_4.back()*(1+growth_rate*pow(fitness_factor,4)*dt-contraction_r
ate*dt)+a_4-a_5-mut_4-c_4+c_5);
```

```
plus_5.push_back(plus_5.back()*(1+growth_rate*pow(fitness_factor,5)*dt-contraction_r
ate*dt)+a_5-a_6-mut_5-c_5);

        plus_0_mut.push_back(plus_0_mut.back()*(1+growth_rate*dt)+mut_0);

plus_1_mut.push_back(plus_1_mut.back()*(1+growth_rate*pow(fitness_factor,1)*dt)+mut_
1);

plus_2_mut.push_back(plus_2_mut.back()*(1+growth_rate*pow(fitness_factor,2)*dt)+mut_
2);

plus_3_mut.push_back(plus_3_mut.back()*(1+growth_rate*pow(fitness_factor,3)*dt)+mut_
3);

plus_4_mut.push_back(plus_4_mut.back()*(1+growth_rate*pow(fitness_factor,4)*dt)+mut_
4);

plus_5_mut.push_back(plus_5_mut.back()*(1+growth_rate*pow(fitness_factor,5)*dt)+mut_
5);
    }
}
```
