# Supplementary material for: Assessing spacer acquisition rates in E. coli type I-E CRISPR arrays
Source: Front Microbiol. 2025 Jan 20;15:1498959. doi: 10.3389/fmicb.2024.1498959 (PMC11788318; doi:10.3389/fmicb.2024.1498959)
Supplement: Supplementary file 3 [file Data_Sheet_2.PDF]

```
//  Supplemental code 4
//  main.cpp
//  cell growth simulation
//
//  Created by 古德猫宁 Harry on 5/21/24.
//

#include <iostream>
#include <vector>
#include <cmath>
//#include <gnuplot-iostream.h>
using namespace std;


void one_day_growth(int date, double growth_rate, double expansion_fraction, double
fitness_factor, double contraction_rate, double acquisition_rate, double mutation,
double red_expansion, vector<double>& plus_0, vector<double>& plus_1,
vector<double>& plus_2, vector<double>& plus_3, vector<double>& plus_4,
vector<double>& plus_5, vector<double>& plus_0_mut, vector<double>& plus_1_mut,
vector<double>& plus_2_mut, vector<double>& plus_3_mut, vector<double>& plus_4_mut,
vector<double>& plus_5_mut, double dt);

void simulation(vector<double>& fraction_p0, vector<double>& fraction_p1,
vector<double>& fraction_p2, vector<double>& fraction_p3, vector<double>&
fraction_p4, vector<double>& fraction_p5, double fit_fac, double exp_frac, double
contraction, double acq_rate, double mut_rate, double acq_red, double dt, double
time_24[], bool fitting);

void residue_calc(vector<double>& fraction_p0, vector<double>& fraction_p1,
vector<double>& fraction_p2, vector<double>& fraction_p3, vector<double>&
fraction_p4, vector<double>& fraction_p5, double exp_p0[], double exp_p1[], double
exp_p2[], double exp_p3[], double exp_p4[], double exp_p5[], double stdev_p0[],
double stdev_p1[], double stdev_p2[], double stdev_p3[], double stdev_p4[], double
stdev_p5[], vector<double>& residue, double dt);

int main(int argc, const char * argv[]) {
    //data for pUC+NHEJ
//    double exp_p0[5] = {93.43438201, 76.40661979, 64.25617988, 54.38759197,
49.19094312};
//    double exp_p1[5] = {6.56561799, 21.25633824, 30.39013717, 35.23328196,
35.92087289};
//    double exp_p2[5] = {0, 2.337041973, 4.732532044, 8.78029872, 12.29130931};
//    double exp_p3[5] = {0, 0, 0.621150908, 1.598827353, 2.596874685};
//    double exp_p4[5] = {0, 0, 0, 0, 0};
//    double exp_p5[5] = {0, 0, 0, 0, 0};
//
//    double stdev_p0[5] = {0, 0.385932323, 1.367486226, 0.51173122, 1.832873975};
//    double stdev_p1[5] = {0, 0.186675831, 1.618603133, 0.487926331, 0.897967518};
//    double stdev_p2[5] = {0, 0.405159917, 0.420011217, 0.041965672, 0.899864019};
//    double stdev_p3[5] = {0, 0, 0.057412517, 0.185961966, 0.228328311};
```

```
//    double stdev_p4[5] = {0, 0, 0, 0, 0};
//    double stdev_p5[5] = {0, 0, 0, 0, 0};

    //data for pUC only
//    double exp_p0[5] = {98.68002189, 88.24568692, 80.1555152, 72.24895971,
66.2536873};
//    double exp_p1[5] = {1.319978108, 11.56674381, 18.41457208, 25.17999081,
29.44573951};
//    double exp_p2[5] = {0, 0.187569274, 1.429912724, 2.571049478, 3.693009428};
//    double exp_p3[5] = {0, 0, 0, 0, 0.607563762};
//    double exp_p4[5] = {0, 0, 0, 0, 0};
//    double exp_p5[5] = {0, 0, 0, 0, 0};
//
//    double stdev_p0[5] = {1.021373287, 2.648646191, 2.831403902, 2.608715256,
2.623898087};
//    double stdev_p1[5] = {1.021373287, 2.565233378, 2.203446487, 1.474986076,
1.558069105};
//    double stdev_p2[5] = {0, 0.218515535, 0.650830978, 1.144575614, 1.262620654};
//    double stdev_p3[5] = {0, 0, 0, 0, 0.351811337};
//    double stdev_p4[5] = {0, 0, 0, 0, 0};
//    double stdev_p5[5] = {0, 0, 0, 0, 0};

    //data for base recording
//    double exp_p0[5] = {100, 94.65565067, 91.72322151, 87.38198513, 85.26517903};
//    double exp_p1[5] = {0, 5.344349333, 8.27677849, 12.16220257, 14.07623757};
//    double exp_p2[5] = {0, 0, 0, 0.455812294, 0.658583399};
//    double exp_p3[5] = {0, 0, 0, 0, 0};
//    double exp_p4[5] = {0, 0, 0, 0, 0};
//    double exp_p5[5] = {0, 0, 0, 0, 0};
//
//    double stdev_p0[5] = {0, 0.842940041, 0.293523492, 0.967835333, 1.509227302};
//    double stdev_p1[5] = {0, 0.842940041, 0.293523492, 0.710440962, 1.622354985};
//    double stdev_p2[5] = {0, 0, 0, 0.395434639, 0.147751616};
//    double stdev_p3[5] = {0, 0, 0, 0, 0};
//    double stdev_p4[5] = {0, 0, 0, 0, 0};
//    double stdev_p5[5] = {0, 0, 0, 0, 0};

    //data for NHEJ
//    double exp_p0[5] = {98.77470518, 93.13165695, 89.84577263, 86.90046253,
81.49807922};
//    double exp_p1[5] = {1.225294819, 6.48514061, 9.515652068, 12.20206851,
17.19105145};
//    double exp_p2[5] = {0, 0.38320244, 0.638575301, 0.897468966, 1.310869331};
//    double exp_p3[5] = {0, 0, 0, 0, 0};
//    double exp_p4[5] = {0, 0, 0, 0, 0};
//    double exp_p5[5] = {0, 0, 0, 0, 0};
//
//    double stdev_p0[5] = {0.140835804, 0.151121757, 0.915769529, 1.371806442,
1.1092972};
//    double stdev_p1[5] = {0.140835804, 0.273931041, 0.977866762, 1.10663971,
```

```
1.218071253};
//    double stdev_p2[5] = {0, 0.124483416, 0.139218888, 0.380567629, 0.125405337};
//    double stdev_p3[5] = {0, 0, 0, 0, 0};
//    double stdev_p4[5] = {0, 0, 0, 0, 0};
//    double stdev_p5[5] = {0, 0, 0, 0, 0};

    //data for pUC only (10 days)
    double exp_p0[10] = {97.79410778, 86.9952406, 76.15969523, 68.50062615,
63.83035072, 56.01534381, 51.37587892, 50.42081054, 51.2314365, 54.67440702};
    double exp_p1[10] = {2.205892222, 12.1219938, 21.87316845, 27.78744509,
30.25634484, 35.33670393, 37.30175587, 37.92434032, 35.91661416, 34.04226296};
    double exp_p2[10] = {0, 0.882765597, 1.967136322, 3.274778113, 5.047020383,
7.481615407, 9.382603573, 9.93099117, 10.52367428, 8.932672742};
    double exp_p3[10] = {0, 0, 0, 0.437150648, 0.866284054, 1.056000278,
1.504567411, 1.46079022, 1.921392698, 1.895378571};
    double exp_p4[10] = {0, 0, 0, 0, 0, 0.110336582, 0.435194228, 0.26306775,
0.406882358, 0.455278701};
    double exp_p5[10] = {0, 0, 0, 0, 0, 0, 0, 0, 0, 0};

    double stdev_p0[10] = {0.669079091, 2.273438148, 1.20091061, 0.824365922,
2.183878841, 3.169963797, 3.738634947, 4.002528157, 7.505720827, 11.64500297};
    double stdev_p1[10] = {0.669079091, 2.190469248, 1.446675011, 0.415013646,
1.604290657, 1.680678951, 1.65448587, 2.267565339, 4.895892063, 8.609498115};
    double stdev_p2[10] = {0, 0.18916373, 0.258716543, 0.274830458, 0.513099038,
1.231095273, 1.724035579, 1.092956764, 1.611052715, 2.118438198};
    double stdev_p3[10] = {0, 0, 0, 0.177164413, 0.22794405, 0.44839837,
0.788591926, 0.54593471, 0.806051537, 1.097601061};
    double stdev_p4[10] = {0, 0, 0, 0, 0, 0.191108566, 0.424487832, 0.246800874,
0.353760603, 0.413060917};
    double stdev_p5[10] = {0, 0, 0, 0, 0, 0, 0, 0, 0, 0};

    double time24_fraction[6] = {exp_p0[0], exp_p1[0], exp_p2[0], exp_p3[0],
exp_p4[0], exp_p5[0]}; //do not change

    double dt = 0.01; //do not change

    double min_fitness_factor = 0.9942;
    double max_fitness_factor = 0.9944;
    double fit_fac_increment = 0.0001; //increment value for each round of fitting
    double fit_fac = 1; //if this variable isn't changing, define it here (1)

    double min_expansion_fraction = 0.824;
    double max_expansion_fraction = 0.832;
    double exp_frac_increment = 0.001;
    double exp_frac = 1; //if this variable isn't changing, define it here (1)

    double min_contraction_rate = 2.52E-4;
    double max_contraction_rate = 2.6E-4;
    double contraction_increment = 0.01E-4;
    double contraction = 0; //if this variable isn't changing, define it here (0)
```

```
    double min_mutation_rate = 0;
    double max_mutation_rate = 0;
    double mut_rate_increment = 0.01E-5;
    double mut_rate = 0; //if this variable isn't changing, define it here (0)

    double min_acquisition_reduction = 0.849;
    double max_acquisition_reduction = 0.851;
    double acq_red_increment = 0.001;
    double acq_red = 0.85; //if this variable isn't changing, define it here (1)

    double min_acquisition_rate = 9.2E-5;
    double max_acquisition_rate = 9.4E-5;
    double acq_rate_increment = 0.02E-5;
    double acq_rate = 9.32E-5; //if this variable isn't changing, define it here

    double min_residue = 10E20;
    double acquisition_reduction = 100; // !!! things to change !!! (if fitting)
    double acquisition_rate = 100;

    bool if_fitting = false; //true if doing a fitting, and false if plotting the
fractions of each array length

    if(if_fitting == false){
        vector<double> fraction_p0;
        vector<double> fraction_p1;
        vector<double> fraction_p2;
        vector<double> fraction_p3;
        vector<double> fraction_p4;
        vector<double> fraction_p5;

        simulation(fraction_p0, fraction_p1, fraction_p2, fraction_p3, fraction_p4,
fraction_p5, fit_fac, exp_frac, contraction, acq_rate, mut_rate, acq_red, dt,
time24_fraction, if_fitting);

        vector<double> residue;

        residue_calc(fraction_p0, fraction_p1, fraction_p2, fraction_p3,
fraction_p4, fraction_p5, exp_p0, exp_p1, exp_p2, exp_p3, exp_p4, exp_p5, stdev_p0,
stdev_p1, stdev_p2, stdev_p3, stdev_p4, stdev_p5, residue, dt);

        cout << " 0: " << residue[0] << " 1: " << residue[1] << " 2: " << residue[2]
<< " 3: " << residue[3] << " 4: " << residue[4] << endl;

        double average_res =
(residue[0]+residue[1]+residue[2]+residue[3]+residue[4])/5; // !!! things to change
!!! divide by the # of nonzero array len

        cout << "average residue: " << average_res << endl;
    }
```

```
    else{
        int iteration = 0;
        for(double acq_red = min_acquisition_reduction; acq_red <=
max_acquisition_reduction; acq_red += acq_red_increment){
            for(double acq_rate = min_acquisition_rate; acq_rate <=
max_acquisition_rate; acq_rate += acq_rate_increment){// !!! things to change !!!
(if fitting)
                iteration++;
            }}
        double loop = 0;

        for(double acq_red = min_acquisition_reduction; acq_red <=
max_acquisition_reduction; acq_red += acq_red_increment){ // !!! things to change
!!! (if fitting)
            for(double acq_rate = min_acquisition_rate; acq_rate <=
max_acquisition_rate; acq_rate += acq_rate_increment){
                loop++;
                int progress = loop/iteration*100;
                cout << progress << "%" << endl;

                vector<double> fraction_p0;
                vector<double> fraction_p1;
                vector<double> fraction_p2;
                vector<double> fraction_p3;
                vector<double> fraction_p4;
                vector<double> fraction_p5;

                simulation(fraction_p0, fraction_p1, fraction_p2, fraction_p3,
fraction_p4, fraction_p5, fit_fac, exp_frac, contraction, acq_rate, mut_rate,
acq_red, dt, time24_fraction, if_fitting);

                vector<double> residue;

                residue_calc(fraction_p0, fraction_p1, fraction_p2, fraction_p3,
fraction_p4, fraction_p5, exp_p0, exp_p1, exp_p2, exp_p3, exp_p4, exp_p5, stdev_p0,
stdev_p1, stdev_p2, stdev_p3, stdev_p4, stdev_p5, residue, dt);

                double average_res =
(residue[0]+residue[1]+residue[2]+residue[3]+residue[4])/5; // !!! things to change
!!! divide by the # of nonzero array len (if fitting)
//                double average_res = residue[0];
                if(average_res < min_residue){
                    min_residue = average_res;
                    acquisition_reduction = acq_red; // !!! things to change !!! (if
fitting)
                    acquisition_rate = acq_rate;
                }
            }
        }
        cout << "minimum residue: " << min_residue << endl;
```

```
        cout << "acquisition reduction: " << acquisition_reduction << endl; // !!!
things to change !!!  (if fitting)
        cout << "acquisition rate: " << acquisition_rate << endl;
    }
}

//no change needed after this line
-------------------------------------------------------------------------------------
-------------------

void residue_calc(vector<double>& fraction_p0, vector<double>& fraction_p1,
vector<double>& fraction_p2, vector<double>& fraction_p3, vector<double>&
fraction_p4, vector<double>& fraction_p5, double exp_p0[], double exp_p1[], double
exp_p2[], double exp_p3[], double exp_p4[], double exp_p5[], double stdev_p0[],
double stdev_p1[], double stdev_p2[], double stdev_p3[], double stdev_p4[], double
stdev_p5[], vector<double>& residue, double dt){

    double residue_p0 = 0;
    for(int i = 0; i < fraction_p0.size(); i++){
        if((i % int(24*60/dt) == 0) && (stdev_p0[i/int(24*60/dt)] != 0)){
            residue_p0 +=
pow((fraction_p0[i]-exp_p0[i/int(24*60/dt)])/stdev_p0[i/int(24*60/dt)], 2);
        }
    }
    residue_p0 += pow((fraction_p0.back()-exp_p0[9])/stdev_p0[9], 2);

    double residue_p1 = 0;
    for(int i = 0; i < fraction_p1.size(); i++){
        if((i % int(24*60/dt) == 0) && (stdev_p1[i/int(24*60/dt)] != 0)){
            residue_p1 +=
pow((fraction_p1[i]-exp_p1[i/int(24*60/dt)])/stdev_p1[i/int(24*60/dt)], 2);
        }
    }
    residue_p1 += pow((fraction_p1.back()-exp_p1[9])/stdev_p1[9], 2);

    double residue_p2 = 0;
    for(int i = 0; i < fraction_p2.size(); i++){
        if((i % int(24*60/dt) == 0) && (stdev_p2[i/int(24*60/dt)] != 0)){
            residue_p2 +=
pow((fraction_p2[i]-exp_p2[i/int(24*60/dt)])/stdev_p2[i/int(24*60/dt)], 2);
        }
    }
    residue_p2 += pow((fraction_p2.back()-exp_p2[9])/stdev_p2[9], 2);

    double residue_p3 = 0;
    for(int i = 0; i < fraction_p3.size(); i++){
        if((i % int(24*60/dt) == 0) && (stdev_p3[i/int(24*60/dt)] != 0)){
            residue_p3 +=
pow((fraction_p3[i]-exp_p3[i/int(24*60/dt)])/stdev_p3[i/int(24*60/dt)], 2);
        }
```

```
    }
    if(stdev_p3[4] != 0){
        residue_p3 += pow((fraction_p3.back()-exp_p3[9])/stdev_p3[9], 2);
    }

    double residue_p4 = 0;
    for(int i = 0; i < fraction_p4.size(); i++){
        if((i % int(24*60/dt) == 0) && (stdev_p4[i/int(24*60/dt)] != 0)){
            residue_p4 +=
pow((fraction_p4[i]-exp_p4[i/int(24*60/dt)])/stdev_p4[i/int(24*60/dt)], 2);
        }
    }
    residue_p4 += pow((fraction_p4.back()-exp_p4[9])/stdev_p4[9], 2);

    double residue_p5 = 0;
//    for(int i = 0; i < fraction_p5.size(); i++){
//        if(i % int(24*60/dt) == 0){
//            residue_p5 += pow(fraction_p5[i]-exp_p5[i/int(24*60/dt)], 2);
//        }
//    }
//    residue_p5 += pow(fraction_p5.back()-exp_p5[4], 2);

    residue.push_back(residue_p0);
    residue.push_back(residue_p1);
    residue.push_back(residue_p2);
    residue.push_back(residue_p3);
    residue.push_back(residue_p4);
    residue.push_back(residue_p5);
}

void simulation(vector<double>& fraction_p0, vector<double>& fraction_p1,
vector<double>& fraction_p2, vector<double>& fraction_p3, vector<double>&
fraction_p4, vector<double>& fraction_p5, double fit_fac, double exp_frac, double
contraction, double acq_rate, double mut_rate, double acq_red, double dt, double
time_24[], bool fitting){

    double expansion_fraction = exp_frac; //fraction of expansion rate for higher
array length (1)
    double fitness_factor = fit_fac; //fitness of expanded clones compared to
unexpanded (1)
    double contraction_rate = contraction; //contraction throughout whole culture
(0)
    double red_expansion = acq_red; //fraction array expansion rate over time (1)

    int cell_count_init = 50000; //number of cells to start with (do not change)
    double growth_rate = 0.02; //growth rate per minute (do not change)
    double acquisition_rate = acq_rate; //array expansion per minute
    double mutation = mut_rate; //rate of mutation per minute(0)

    double timestamp = dt; //minute per loop (60 = 1 hour)
```

```
    int days = 9;

    vector<double> plus_0;
    plus_0.push_back(cell_count_init*time_24[0]);
    vector<double> plus_0_mut;
    plus_0_mut.push_back(0);
    vector<double> plus_1;
    plus_1.push_back(cell_count_init*time_24[1]);
    vector<double> plus_1_mut;
    plus_1_mut.push_back(0);
    vector<double> plus_2;
    plus_2.push_back(cell_count_init*time_24[2]);
    vector<double> plus_2_mut;
    plus_2_mut.push_back(0);
    vector<double> plus_3;
    plus_3.push_back(cell_count_init*time_24[3]);
    vector<double> plus_3_mut;
    plus_3_mut.push_back(0);
    vector<double> plus_4;
    plus_4.push_back(cell_count_init*time_24[4]);
    vector<double> plus_4_mut;
    plus_4_mut.push_back(0);
    vector<double> plus_5;
    plus_5.push_back(cell_count_init*time_24[5]);
    vector<double> plus_5_mut;
    plus_5_mut.push_back(0);

    for(int i = 0; i < days; i++){ //i is number of days
        one_day_growth(i, growth_rate, expansion_fraction, fitness_factor,
contraction_rate, acquisition_rate, mutation, red_expansion, plus_0, plus_1, plus_2,
plus_3, plus_4, plus_5, plus_0_mut, plus_1_mut, plus_2_mut, plus_3_mut, plus_4_mut,
plus_5_mut, dt);

        double total_cell =
plus_0.back()+plus_1.back()+plus_2.back()+plus_3.back()+plus_4.back()+plus_5.back()+
plus_0_mut.back()+plus_1_mut.back()+plus_2_mut.back()+plus_3_mut.back()+plus_4_mut.b
ack()+plus_5_mut.back();
        double innoculate_fraction = 50000/total_cell;

        //cout << "innoculate_fraction" << innoculate_fraction << endl;

        plus_0.back() *= innoculate_fraction;
        plus_0_mut.back() *= innoculate_fraction;
        plus_1.back() *= innoculate_fraction;
        plus_1_mut.back() *= innoculate_fraction;
        plus_2.back() *= innoculate_fraction;
        plus_2_mut.back() *= innoculate_fraction;
        plus_3.back() *= innoculate_fraction;
        plus_3_mut.back() *= innoculate_fraction;
        plus_4.back() *= innoculate_fraction;
```

```
        plus_4_mut.back() *= innoculate_fraction;
        plus_5.back() *= innoculate_fraction;
        plus_5_mut.back() *= innoculate_fraction;
    }


    for(int i = 0; i < days*1440/dt; i++){
        double tot_cell =
plus_0[i]+plus_1[i]+plus_2[i]+plus_3[i]+plus_4[i]+plus_5[i]+plus_0_mut[i]+plus_1_mut
[i]+plus_2_mut[i]+plus_3_mut[i]+plus_4_mut[i]+plus_5_mut[i];
        //cout << "total cells: " << tot_cell << endl;
        fraction_p0.push_back((plus_0[i]+plus_0_mut[i])/tot_cell*100);
        fraction_p1.push_back((plus_1[i]+plus_1_mut[i])/tot_cell*100);
        fraction_p2.push_back((plus_2[i]+plus_2_mut[i])/tot_cell*100);
        fraction_p3.push_back((plus_3[i]+plus_3_mut[i])/tot_cell*100);
        fraction_p4.push_back((plus_4[i]+plus_4_mut[i])/tot_cell*100);
        fraction_p5.push_back((plus_5[i]+plus_5_mut[i])/tot_cell*100);

    }
    //cout << "fraction_p0 size: " << fraction_p0.size() << endl;
    if(fitting == false){
        cout << "fraction_p0: " << endl;
        for(int i = 0; i < fraction_p0.size(); i++){
            if(i % int(24*60/dt) == 0){
                cout << fraction_p0[i] << endl;
            }
        }
        cout << fraction_p0.back() << endl;
        cout << "fraction_p1: " << endl;
        for(int i = 0; i < fraction_p1.size(); i++){
            if(i % int(24*60/dt) == 0){
                cout << fraction_p1[i] << endl;
            }
        }
        cout << fraction_p1.back() << endl;
        cout << "fraction_p2: " << endl;
        for(int i = 0; i < fraction_p2.size(); i++){
            if(i % int(24*60/dt) == 0){
                cout << fraction_p2[i] << endl;
            }
        }
        cout << fraction_p2.back() << endl;
        cout << "fraction_p3: " << endl;
        for(int i = 0; i < fraction_p3.size(); i++){
            if(i % int(24*60/dt) == 0){
                cout << fraction_p3[i] << endl;
            }
        }
        cout << fraction_p3.back() << endl;
        cout << "fraction_p4: " << endl;
```

```
        for(int i = 0; i < fraction_p4.size(); i++){
            if(i % int(24*60/dt) == 0){
                cout << fraction_p4[i] << endl;
            }
        }
        cout << fraction_p4.back() << endl;
        cout << "fraction_p5: " << endl;
        for(int i = 0; i < fraction_p5.size(); i++){
            if(i % int(24*60/dt) == 0){
                cout << fraction_p5[i] << endl;
            }
        }
        cout << fraction_p5.back() << endl;
    }
}

void one_day_growth(int date, double growth_rate, double expansion_fraction, double
fitness_factor, double contraction_rate, double acquisition_rate, double mutation,
double red_expansion, vector<double>& plus_0, vector<double>& plus_1,
vector<double>& plus_2, vector<double>& plus_3, vector<double>& plus_4,
vector<double>& plus_5, vector<double>& plus_0_mut, vector<double>& plus_1_mut,
vector<double>& plus_2_mut, vector<double>& plus_3_mut, vector<double>& plus_4_mut,
vector<double>& plus_5_mut, double dt){
    //cout << cell_count_init << rep_period << expansion_fraction << fitness_factor
<< contraction_rate << endl;
    int timepoint = int(1440/dt);
//    cout << "timepoint: " << timepoint << endl;

    for(int i = 1; i <= timepoint; i++){
//        cout << "date: " << date;
//        cout << "in if: date: " << date << "i: " << i << endl;
        double a_1 = plus_0.back()*acquisition_rate*dt*pow(red_expansion, date);
//inflow to plus 1 without contraction or outflow to plus 2
        double a_2 =
plus_1.back()*acquisition_rate*expansion_fraction*dt*pow(red_expansion, date);
        double a_3 = plus_2.back()*acquisition_rate*pow(expansion_fraction,
2)*dt*pow(red_expansion, date);
        double a_4 = plus_3.back()*acquisition_rate*pow(expansion_fraction,
3)*dt*pow(red_expansion, date);
        double a_5 = plus_4.back()*acquisition_rate*pow(expansion_fraction,
4)*dt*pow(red_expansion, date);
        double a_6 = plus_5.back()*acquisition_rate*pow(expansion_fraction,
5)*dt*pow(red_expansion, date);

        double c_1 = plus_1.back()*contraction_rate*dt;
        double c_2 = plus_2.back()*contraction_rate*dt;
        double c_3 = plus_3.back()*contraction_rate*dt;
        double c_4 = plus_4.back()*contraction_rate*dt;
        double c_5 = plus_5.back()*contraction_rate*dt;
```

```
        double mut_0 = plus_0.back()*mutation*dt;
        double mut_1 = plus_1.back()*mutation*dt;
        double mut_2 = plus_2.back()*mutation*dt;
        double mut_3 = plus_3.back()*mutation*dt;
        double mut_4 = plus_4.back()*mutation*dt;
        double mut_5 = plus_5.back()*mutation*dt;

        //fitness factor start at plus 2, inflow/outflow first then grow
//
plus_0.push_back((plus_0.back()-temp_1-mut_0)*(1+growth_rate*dt-contraction_rate*dt)
);
//
plus_1.push_back((plus_1.back()+temp_1-temp_2-mut_1)*(1+growth_rate*dt-contraction_r
ate*dt));
//
plus_2.push_back((plus_2.back()+temp_2-temp_3-mut_2)*(1+growth_rate*pow(fitness_fact
or,1)*dt-contraction_rate*dt));
//
plus_3.push_back((plus_3.back()+temp_3-temp_4-mut_3)*(1+growth_rate*pow(fitness_fact
or,2)*dt-contraction_rate*dt));
//
plus_4.push_back((plus_4.back()+temp_4-temp_5-mut_4)*(1+growth_rate*pow(fitness_fact
or,3)*dt-contraction_rate*dt));
//
plus_5.push_back((plus_5.back()+temp_5-temp_6-mut_5)*(1+growth_rate*pow(fitness_fact
or,4)*dt-contraction_rate*dt));
//
//        plus_0_mut.push_back((plus_0_mut.back()+mut_0)*(1+growth_rate*dt));
//        plus_1_mut.push_back((plus_1_mut.back()+mut_1)*(1+growth_rate*dt)+mut_1);
//
plus_2_mut.push_back((plus_2_mut.back()+mut_2)*(1+growth_rate*pow(fitness_factor,1)*
dt));
//
plus_3_mut.push_back((plus_3_mut.back()+mut_3)*(1+growth_rate*pow(fitness_factor,2)*
dt));
//
plus_4_mut.push_back((plus_4_mut.back()+mut_4)*(1+growth_rate*pow(fitness_factor,3)*
dt));
//
plus_5_mut.push_back((plus_5_mut.back()+mut_5)*(1+growth_rate*pow(fitness_factor,4)*
dt));

        //fitness factor start at plus 1, grow then inflow/outflow

plus_0.push_back(plus_0.back()*(1+growth_rate*dt-contraction_rate*dt)-a_1-mut_0+c_1)
;

plus_1.push_back(plus_1.back()*(1+growth_rate*pow(fitness_factor,1)*dt)+a_1-a_2-mut_
1-c_1+c_2);
```

```
plus_2.push_back(plus_2.back()*(1+growth_rate*pow(fitness_factor,2)*dt-contraction_r
ate*dt)+a_2-a_3-mut_2-c_2+c_3);

plus_3.push_back(plus_3.back()*(1+growth_rate*pow(fitness_factor,3)*dt-contraction_r
ate*dt)+a_3-a_4-mut_3-c_3+c_4);

plus_4.push_back(plus_4.back()*(1+growth_rate*pow(fitness_factor,4)*dt-contraction_r
ate*dt)+a_4-a_5-mut_4-c_4+c_5);

plus_5.push_back(plus_5.back()*(1+growth_rate*pow(fitness_factor,5)*dt-contraction_r
ate*dt)+a_5-a_6-mut_5-c_5);

        plus_0_mut.push_back(plus_0_mut.back()*(1+growth_rate*dt)+mut_0);

plus_1_mut.push_back(plus_1_mut.back()*(1+growth_rate*pow(fitness_factor,1)*dt)+mut_
1);

plus_2_mut.push_back(plus_2_mut.back()*(1+growth_rate*pow(fitness_factor,2)*dt)+mut_
2);

plus_3_mut.push_back(plus_3_mut.back()*(1+growth_rate*pow(fitness_factor,3)*dt)+mut_
3);

plus_4_mut.push_back(plus_4_mut.back()*(1+growth_rate*pow(fitness_factor,4)*dt)+mut_
4);

plus_5_mut.push_back(plus_5_mut.back()*(1+growth_rate*pow(fitness_factor,5)*dt)+mut_
5);
    }
}
```
