# Supplementary material for: Assessing spacer acquisition rates in E. coli type I-E CRISPR arrays
Source: Front Microbiol. 2025 Jan 20;15:1498959. doi: 10.3389/fmicb.2024.1498959 (PMC11788318; doi:10.3389/fmicb.2024.1498959)
Supplement: Supplementary file 5 [file Data_Sheet_4.PDF]

```
% Supplemental code 2

clear all
close all
% rate of spacer acquisition dS/dt = r * Ni
% cells grow at rate dNi/dt = mu * Ni
% cells grow from 10^4 cells to 10^8 cells and then are transferred

% general parameters
No = 10^4; % number of cells inoculated into each tube
Nf = 10^8; %number of cells at a cell transfer event
r = 8.16833E-05; % array expansion rate per cell per minute
mu = 0.02; % in 1/min
mut = 10^-6; % mutation rate of Cas12 deactivation per minute per cell
max_time  =  14400; % maximum time in minutes
time_points = 2880; % number of time steps
dt = max_time / time_points;
max_array = 21; % maximum extension of the array - 1 (array length 1 = no extension)

% start at 24 h
t_min =24*60/dt;

% change in growth rate constant of Cas12 mutants
mut_fit = 1.03;
% parameters for modified model
mu_red = 1.00; %cell growth rate can be reduced for cells with expanded arrays
r_red = 1.00; %array expansion rate can be reduced for cells with expanded arrays


% colors and markers for plotting
C = {'ok','db','sr','xg','+c'};
day_counter = 1;

% cells stores the number of cells at each array length
% cell(i) is information about the cells with array length i-1 (i=1 is
% unexpanded)
% cells.array is the array length in number of new spacers
% with 0 being the original length
% cells.num is the number of cells with that array length
% there is a separate structure that keeps track of cells with mutated
% Cas machinery, these cells have an expansion rate of 0

% keep track of cells with functional Cas12 proteins
for j = 1:max_array
cells(j).array = j-1;
cells(j).num = 0;
cells(j).growth = mu*mu_red^(j-1);
cells(j).r = r*r_red^(j-1);
end
```

```
% keep track of mutated cells that may grow faster but array expansion
% stops
for j = 1:max_array
cells_mut(j).array = j-1;
cells_mut(j).num = 0;
% x% fitness advantage for cells with non-functional Cas12
cells_mut(j).growth = mu*mut_fit;
cells_mut(j).r = 0;
end

% at 24 h start with 6.5% +1 cells and 93.5% +0 cells
cells(1).num = No*0.978;
cells(2).num = No*0.022;

for t = t_min:time_points+1 % time in minutes
    % does an extension occur
    for k = 1:length(cells)
        cell_flips = rand(floor(cells(k).num),1);
        number_ext = sum(cell_flips<(cells(k).r*dt));
        if number_ext > 0
            array_bin = cells(k).array+2;
            cells(array_bin).num = cells(array_bin).num+number_ext;
            cells(k).num = cells(k).num - number_ext;
        end

    % Cas12 mutations
        cell_flips = rand(floor(cells(k).num),1);
        number_mut = sum(cell_flips<(mut*dt));
        if number_mut > 0
            array_bin = cells(k).array+1;
            cells(array_bin).num = cells(array_bin).num-number_mut;
            cells_mut(k).num = cells_mut(k).num + number_mut;
        end

    % the cells grow
    cells(k).num = cells(k).num + cells(k).growth*cells(k).num*dt;
    cells_mut(k).num = cells_mut(k).num + cells_mut(k).growth*cells_mut(k).num*dt;
    end

% transfer cells to a new tube if the population exceeds maximum
tot_cells = floor(sum([cells.num]))+floor(sum([cells_mut.num]));

if tot_cells>Nf

% calculate the fraction of the population for each array length
for m = 1:length(cells)
cells(m).fract = cells(m).num/tot_cells;
cells_mut(m).fract = cells_mut(m).num/tot_cells;
end
```

```
fraction = [0, [cells.fract],[cells_mut.fract]];

% cells are partitioned by picking No random numbers, each random number
% assigns 1 cells to a array length bin
    cell_partition = rand(No,1);
    for m = 1:(length(cells)+length(cells_mut))
% find how many random numbers bewteen certain values
        lower_end = cell_partition>sum(fraction(1:m));
        upper_end = cell_partition <= sum(fraction(1:m+1));
        combined = and(lower_end, upper_end);
        transf_cells = sum(combined);
        if m <= length(cells)
        cells(m).num = transf_cells;
        else
        cells_mut(m-length(cells)).num = transf_cells;
        end

    end

end

% show make a few plots after every 24 h
if mod((t-1)*dt,1440)==0

tot_cells = floor(sum([cells.num]));

% calculate the fraction of cells at each array length
for m = 1:length(cells)
cells(m).fract = cells(m).num/tot_cells;
cells_mut(m).fract = cells_mut(m).num/tot_cells;
band(m) = cells(m).fract+cells_mut(m).fract;
end

% next line calculates the average array length
av_array_length = sum([0:1:max_array-1] * band');

% plot average array length over time
figure(1)
hold on
plot(t*dt/(24*60), av_array_length,'ko')
hold off
xlabel('time / day')
ylabel('average array length')

% % plot the expanded fraction over time
figure(2)
hold on
plot(t*dt/(24*60), sum(band(2:end)),'bo')
hold off
xlabel('time / day')
```

```
ylabel('percent expanded')

% % plot the fraction of cells at each length over time
figure(3)
hold on
for j = 1:5
plot(t*dt/(24*60), cells(j).fract+cells_mut(j).fract, C{j})
end
legend('+0', '+1', '+2', '+3', '+4')
hold off
xlabel('time / day')
ylabel('fraction of population')

% plot fraction of population with mutated Cas12
figure(4)
hold on
plot(t*dt/(24*60), sum([cells_mut.num])/tot_cells,'kx')
hold off
xlabel('time / day')
ylabel('mutant fraction')

% save the data for each 24 h period
data_out(day_counter).time = (t-1)*dt/(24*60);
data_out(day_counter).av_array_length = av_array_length;
data_out(day_counter).per_exp = sum(band(2:end));
data_out(day_counter).per_zeros = cells(1).fract;
data_out(day_counter).per_ones = cells(2).fract;
data_out(day_counter).per_twos = cells(3).fract;
data_out(day_counter).per_three = cells(4).fract;
data_out(day_counter).per_four = cells(5).fract;

data_out(day_counter).per_zeros_mut = cells_mut(1).fract;
data_out(day_counter).per_ones_mut = cells_mut(2).fract;
data_out(day_counter).per_twos_mut = cells_mut(3).fract;
data_out(day_counter).per_three_mut = cells_mut(4).fract;
data_out(day_counter).per_four_mut = cells_mut(5).fract;

day_counter = day_counter+1;

end

end
```
