# Supplementary material for: Assessing spacer acquisition rates in E. coli type I-E CRISPR arrays
Source: Front Microbiol. 2025 Jan 20;15:1498959. doi: 10.3389/fmicb.2024.1498959 (PMC11788318; doi:10.3389/fmicb.2024.1498959)
Supplement: Supplementary file 6 [file Data_Sheet_5.PDF]

Supplementary Material

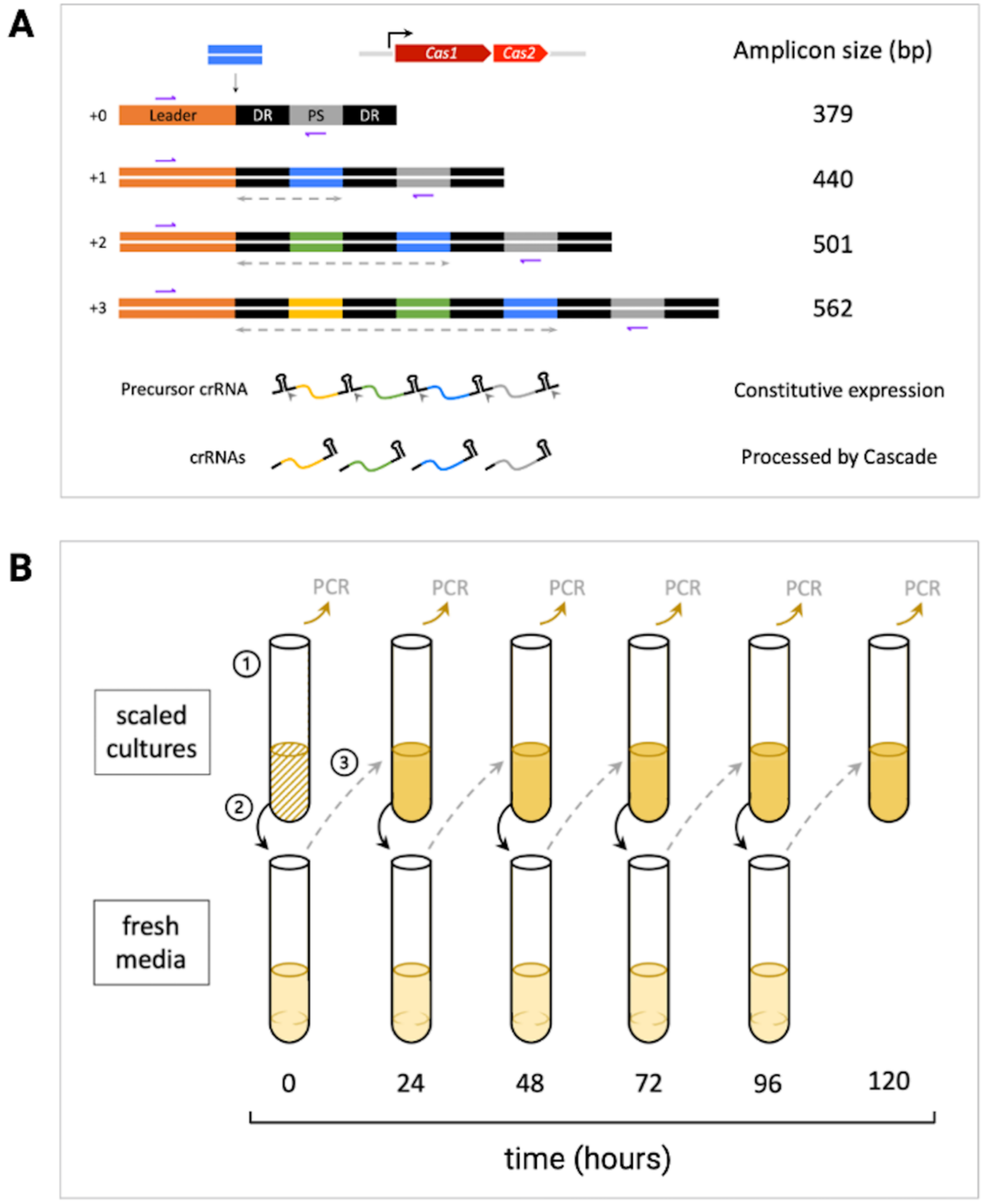

Supplementary Figure 1. Spacer acquisition assay. (**A**) Cas1-Cas2 integrase complex captures and processes short DNA sequences prior to CRISPR-array integration. Arrays contain alternating repeats (black) and spacers. Expanded arrays can be detected using PCR with primers (arrows) that span the spacer integration site (leader-repeat1 junction). Arrays are expressed from a promoter in the leader sequence (orange), producing precursor crRNA, which can be further processed to crRNAs via Cascade (*E. coli*). DR: Direct Repeat; PS: Parental Spacer. (**B**) Five-day Cas1-Cas2 induction time course. 1. Spacer recording strain scaled up overnight at 37ºC with no Cas1-Cas2 induction. 2. Scaled overnight culture used to inoculate fresh media containing induction chemicals and antibiotic(s). 3. After 24 hours, cultures are sampled for PCR to measure the extent of spacer acquisition. Culture is then passaged 1:100 into fresh induction media for a subsequent round of growth. This cycle is repeated for five days.

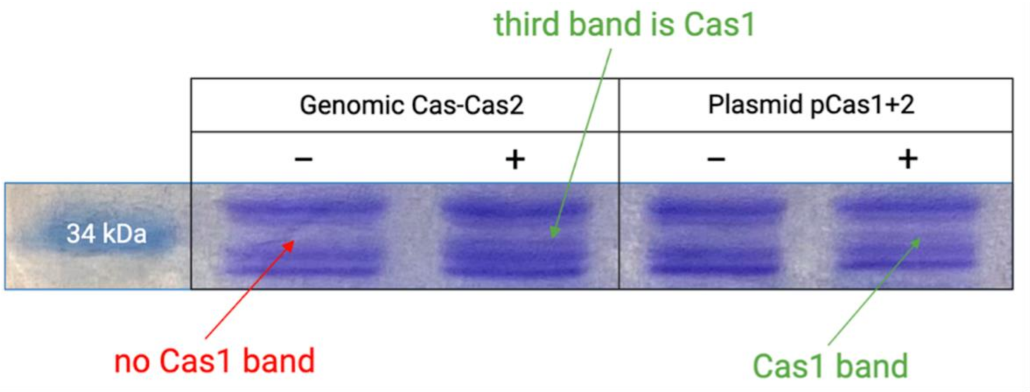

Supplementary Figure 2. Cas1 protein expression confirmed via SDS-PAGE gel. The *E. coli* Type I-E CRISPR Cas1 nuclease is 33.194 kDa. Cas1 expression was confirmed from two sources: A strain containing a single genomic copy of the Cas1 gene in an operon with Cas2 and controlled by a T7-lac promoter (left) and a strain containing a plasmid with a Cas1-Cas2 operon controlled by a T7-lac promoter (right). Both instances show a clear protein band at the Cas1 size upon arabinose and IPTG induction. The genomic construct was derived from the plasmid. Expression was induced with 0.2% arabinose and 1mM IPTG.

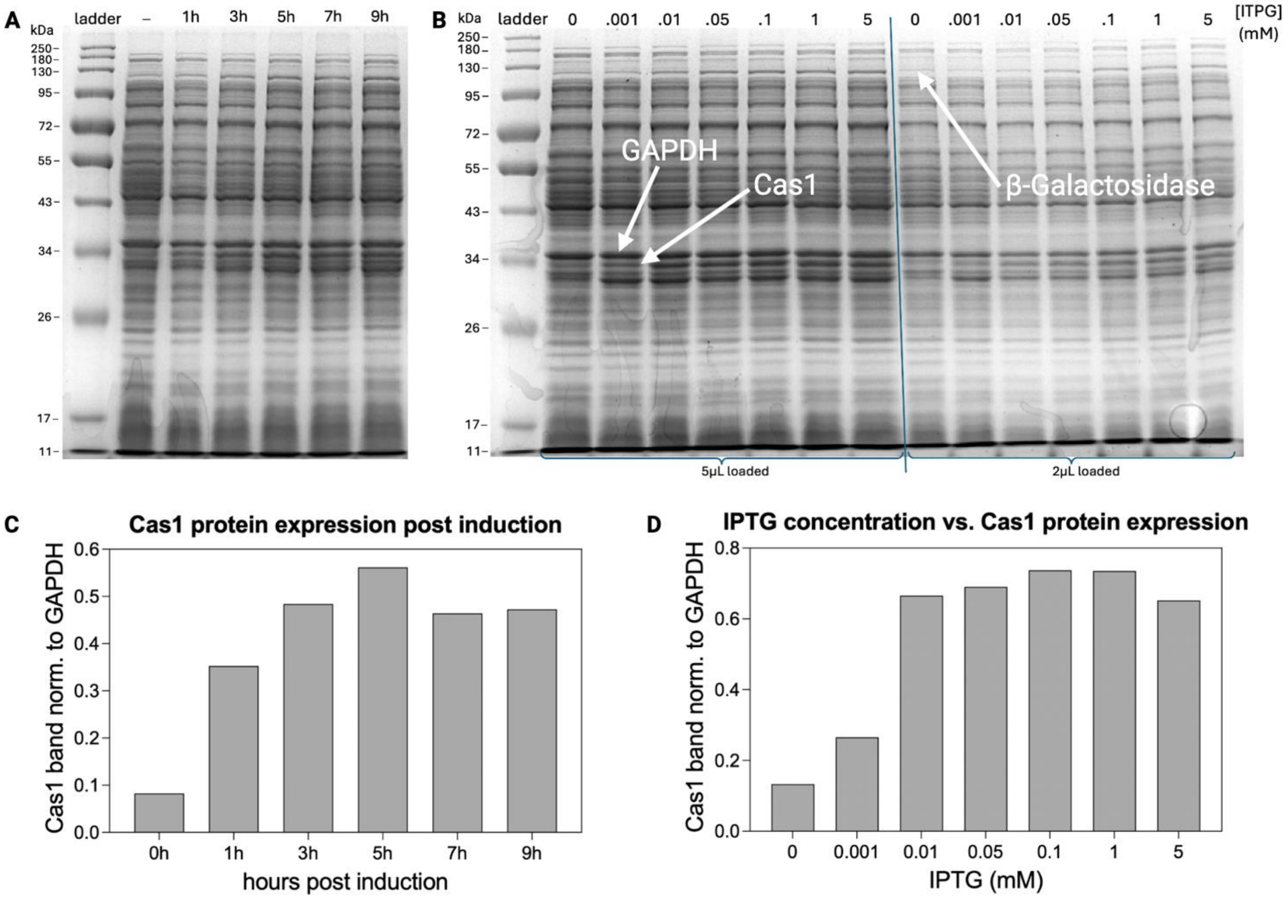

Supplementary Figure 3. Cas1 protein expression vs. time and IPTG concentration. (**A**) Total cell lysate from the base recording strain dosed with IPTG (0.05 mM) and arabinose (0.2%). Lysate was harvested from cultures at distinct time intervals post induction with proteins separated using SDS-PAGE gel electrophoresis. (**B**) Total cell lysate from the base recording strain 3h post induction with fixed arabinose dose (0.2%) and variable IPTG dose (0-5 mM). The 0mM IPTG condition also did not receive arabinose. The Cas1 band is ~33 kDa. Band intensities were quantified and normalized using housekeeping protein GAPDH (~35.5 kDa). LacZ gene product β-Galactosidase is also identified as expression of this protein is upregulated in the presence of IPTG. (**C**) Cas1 protein band intensities quantified and normalized from A. (**D**) Cas1 protein band intensities quantified and normalized from the last 7 lanes of panel B.

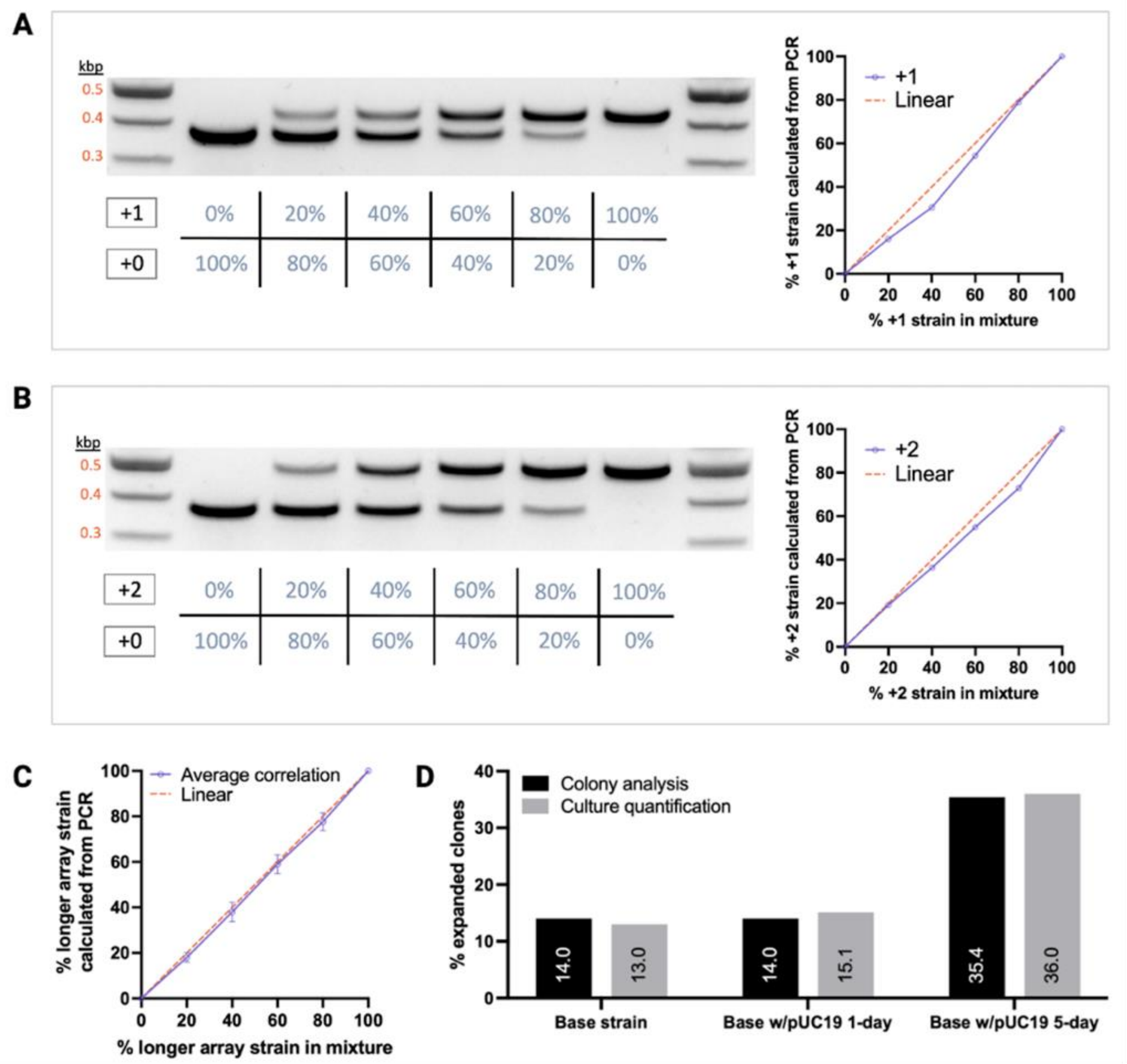

Supplementary Figure 4. Validating the PCR-based assay to measure proportions of array lengths within a population of cells. (**A**) Clonal strains with different array lengths were grown separately, mixed in a known ratio, and analyzed via PCR and gel electrophoresis. Here, strains with arrays of length +0 and +1 were mixed. PCR products were separated by gel electrophoresis and imaged. Image analysis using the ladder bands convert band intensities to picomoles. Correlation curves were generated with known proportions charted versus pmol proportions calculated from the PCR-gel images. (**B**) Clonal +0 and +2 cultures were mixed, processed, and evaluated as in A. (**C**) The average correlation from 15 different mixture series is plotted ±SD. These mixture series were made up of the following pairs: +0 /+1, +0/+2, +1/+2, +3/+5, and +4/+5. Each mixture was run in triplicate producing 15 correlation curves. (**D**) The base recording strain with and without pUC19, starting at array length +0 were grown for either 1 or 5 days with Cas1-Cas2 induction generating a mixed population with multiple array lengths. Cells were isolated by plating on LB-agar, and the array length for individual, clonal isolates was measured using PCR. The fraction of screened clones with an expanded array was compared to the expanded fraction calculated from the culture using the PCR method. 50 colonies were screened in the no-plasmid strain (5-day), 100 in the strain with pUC19 (1-day) and 48 (5-day). These results show error in array-length percentages (as measured using PCR) of ±3.62% on average.

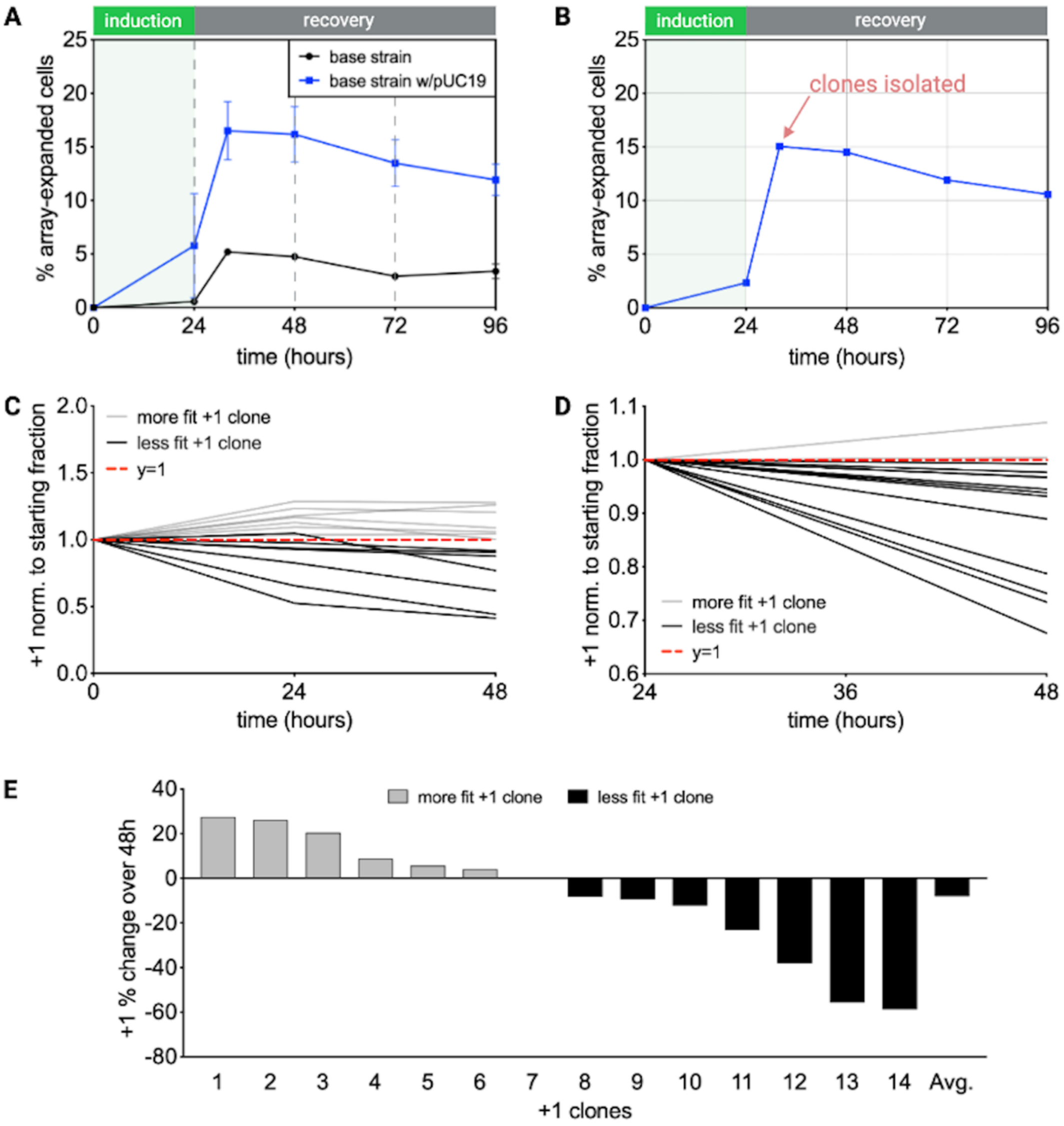

Supplementary Figure 5. Expanded vs. unexpanded-array competition experiments without Cas1-Cas2 induction. (**A**) Two strains, the base recording strain with and without pUC19, were induced for Cas1-Cas2 expression for 24h. Cultures were then passed into fresh media without induction chemicals. Cultures were grown for 8h (24-32h) to allow for residual Cas1-Cas2 to degrade. From 32-96h, PCR measurements identified changing expanded proportions. Vertical dashed lines represent 1:100 passaging into fresh media. (**B**) Clones from one of the replicates (base strain w/pUC19) were isolated from the 32h time point. 100 clones were screened across the leader proximal end of the array, with 14 identified as expanded (all +1). (**C**) Competition experiments were run for 48h competing each of the 14 expanded clones individually with +0. PCR bands were quantified to determine the ratios of +1/+0 at each time point (**D**) +1 proportion changes from the 14 competition experiments across the 24-48h period showing 12 of the fourteen +1 clones losing in proportion to the +0 cells. (**E**) Percent change in the +1 proportion for each of the fourteen +1 clones from the respective competition experiments, from 0-48h. “less/more fit +1 clone” labels refer to +1 fitness relative to the competing +0 population.

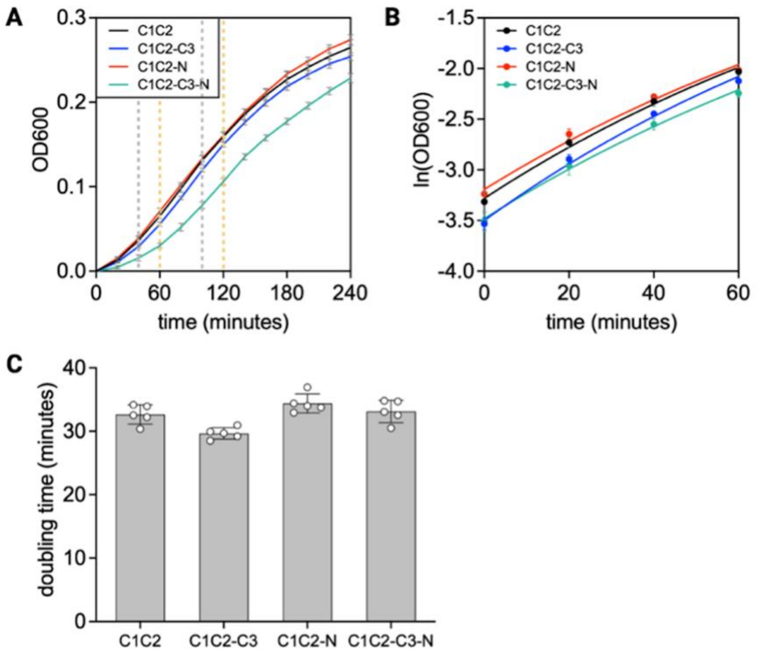

Supplementary Figure 6. Doubling times calculated from OD600 growth curves. OD600 growth curves were generated from 200uL cultures in 96-well plates at 37ºC measured every 20 minutes. (**A**) Log phase growth defined for each of four strains grown in LB media, measured in a plate reader. Vertical gray lines define the log phase growth for strains C1C2, C1C2-C3 and C1C2-N. The orange vertical line defines the log phase growth for strain C1C2-C3-N. (**B**) For each strain log-growth is fit to an exponential growth curve. The x-axis is time post log phase start. (**C**) Doubling times (Td) were calculated from the log growth fits. The fits are of the form $Y=Ae^{Bx}$, where B is the exponential growth rate. Doubling time Td = ln(2)/B.

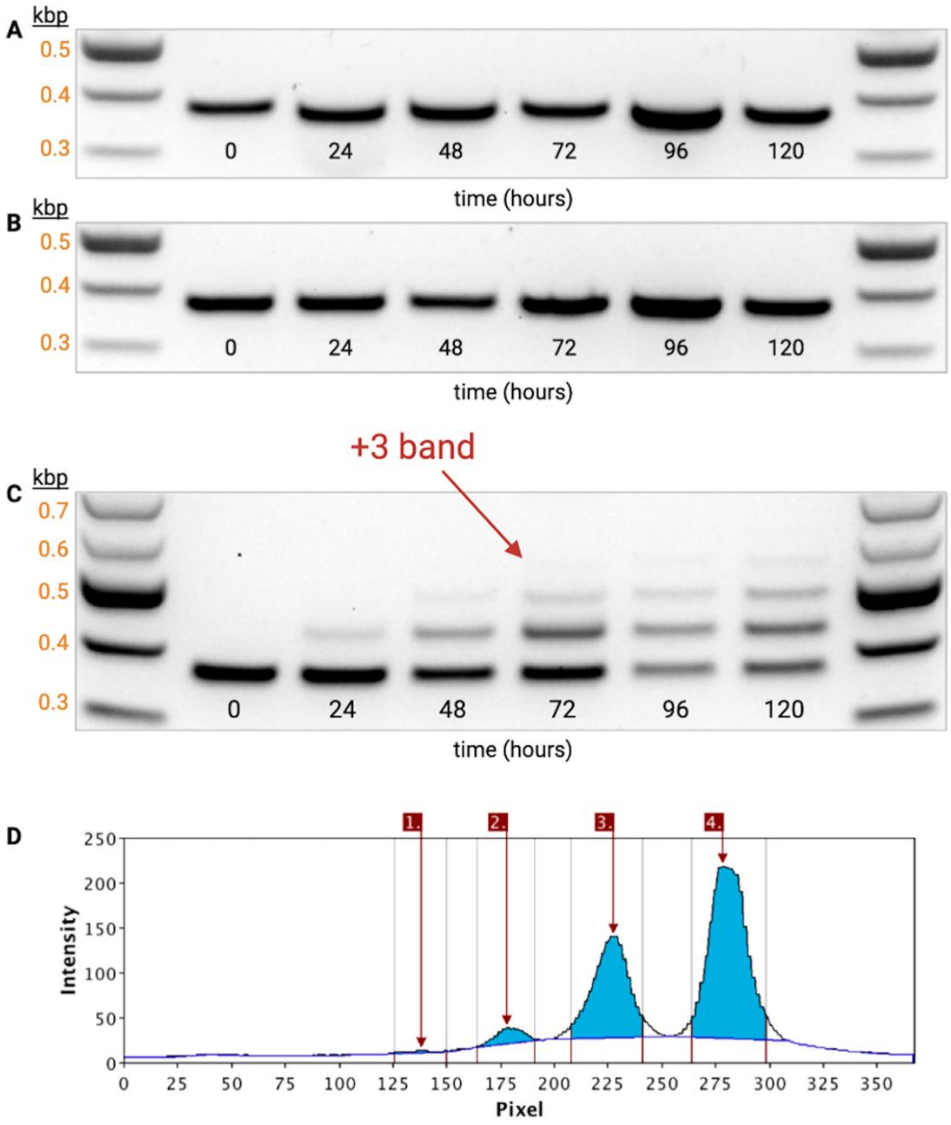

Supplementary Figure 7. Quantifying spacer acquisition using PCR. (**A**) Base recording strain serial passaged for five days without IPTG or arabinose, sampled daily for PCR analysis. No array expansion detected. (**B**) Base recording strain with pUC19 also shows no detectable array expansion through a five-day serial passaging experiment without Cas1-Cas2 induction. (**C**) Minimal population proportion detection using this method is ~0.5%, as seen here for the +3 band at 72h for a spacer recording strain. (**D**) The 72h bands from C are quantified using GelAnalyzer software. The +3 band as indicated by the "1." tag represents 0.57% of the population.

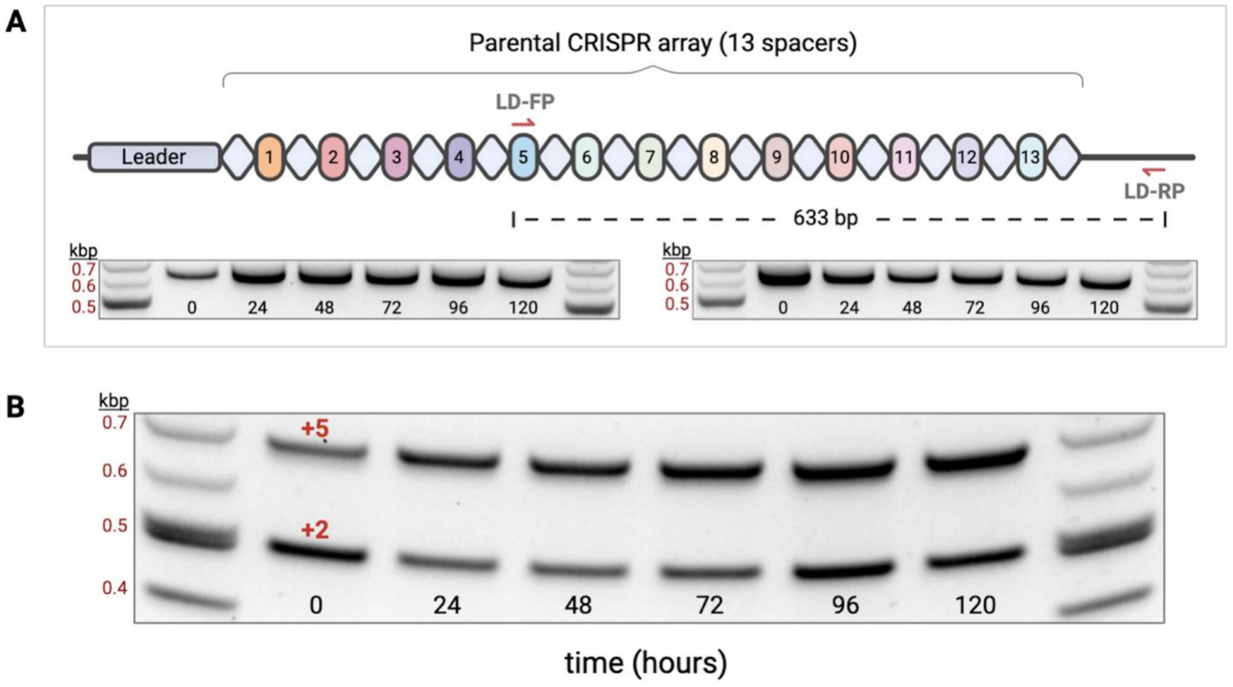

Supplementary Figure 8. Array contraction not detected through 5-days of culturing. (**A**) 5-day Cas1-Cas2 induction experiments using PCR primers spanning the array-distal end of the CRISPR array from parental spacer 5 indicated no loss of spacers (array contraction) in that region. Two strains were monitored (using primers LD-FP and LD-RP), the base recording strain with (right) and without (left) pUC19. Colored parental spacers are numbered 1-13. (**B**) Five expanded-array clones were isolated at the end of a 5-day induction time course containing between +1 and +5 new spacers (base recording strain w/pUC19). These array-expanded strains were mixed in different combinations for a 5-day time course with no Cas1-Cas2 induction. The leader-proximal PCRs for these strains revealed no detectable bands below the parental bands through these experiments.

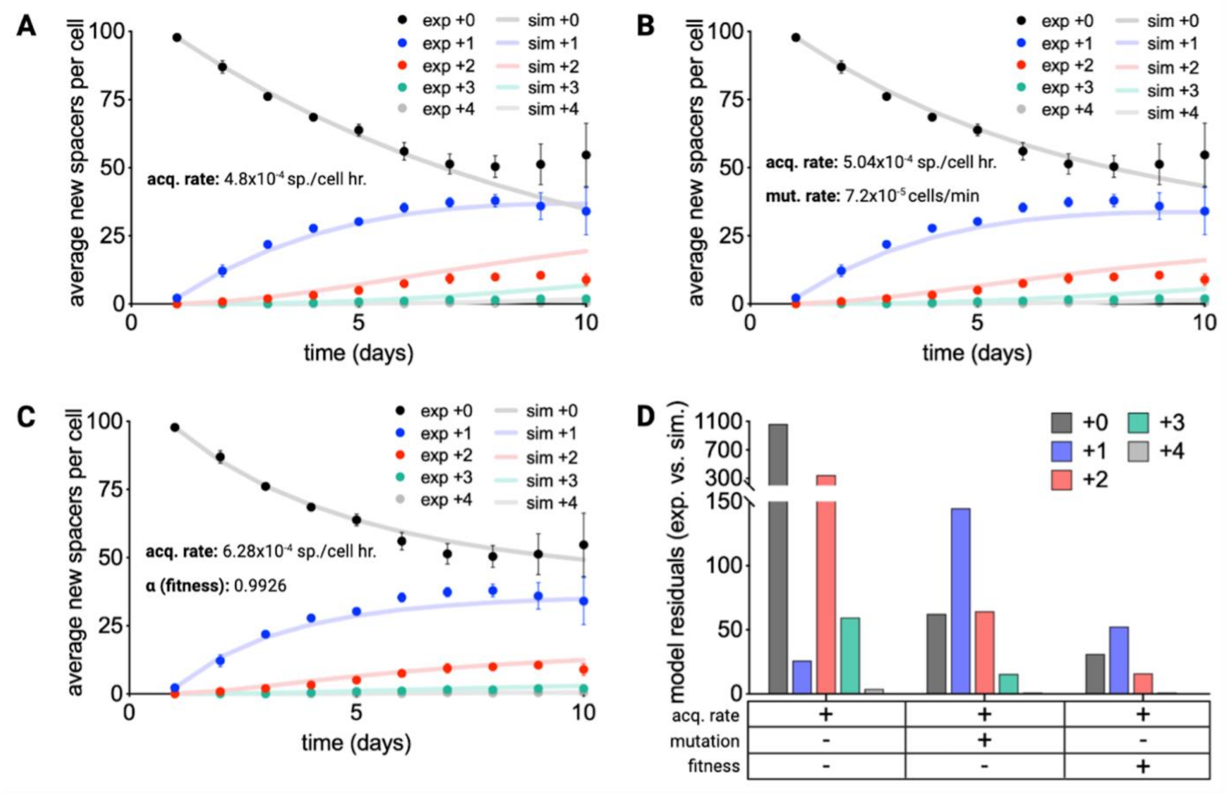

Supplementary Figure 9. Model simulations fitting to a 10-day Cas1-Cas2 induction experiment for the base recording strain containing pUC19. (**A**) The expansion of the array was simulated starting with 100% of the population being at length +0 at t=0. Array expansion, using the rate derived from +0 parental population decay was used to simulate the expansion of all array-length populations over ten days. (**B**) Using a best fit to all array length populations, finding the acquisition rate and Cas1-Cas2 mutation rate that together produce the best fit. Mutation did affect the cell growth rate. (**C**) Best fit model for acquisition rate and fitness, using α^{i} growth for array-length populations where *i* is the number of new spacers. (**D**) Residuals calculated from the model fit to experimental data for each detected expanded-array population. Experimental data is the mean of three biological replicates ±SD. The code for running these simulations is Sim. Code 1. Fits minimize average residual across all array lengths (+0 to +4) over all time points.

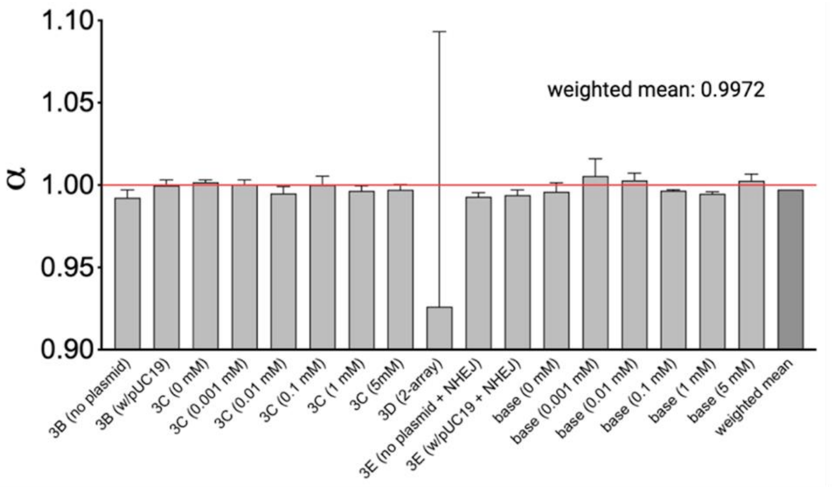

Supplementary Figure 10. Alpha values calculated from five-day Cas1-Cas2 induction experiments. Alpha (α_μ) is the growth penalty for array-expanded populations. The growth rate for a strain decreases by alpha for each new spacer added to the array, as shown in Equation 7. These alpha values were derived from the experiments presented in Figures 3 and S11. Weighted mean alpha for all conditions was 0.9972, calculated using a weight of 1/SD. Error bars represent the SD from bootstrapping based on three experimental replicates.

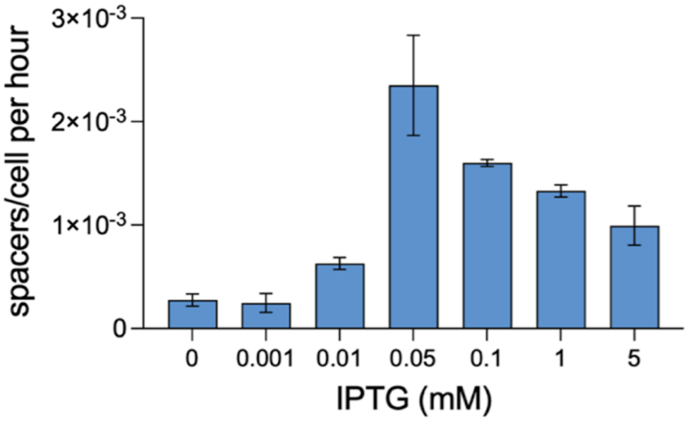

Supplementary Figure 11. Cas1-Cas2 expression range in base spacer recording strain. Modulating Cas1-Cas2 expression by varying the IPTG dose and measuring the corresponding spacer acquisition rates for the base recording strain containing no plasmids. The array expansion rate was calculated as in Figure 3, assuming a fitness cost for array expansion. The array expansion rate is independent of array length. Means of three biological replicates ±SD is reported. *P* values are provided in SI Table 4.

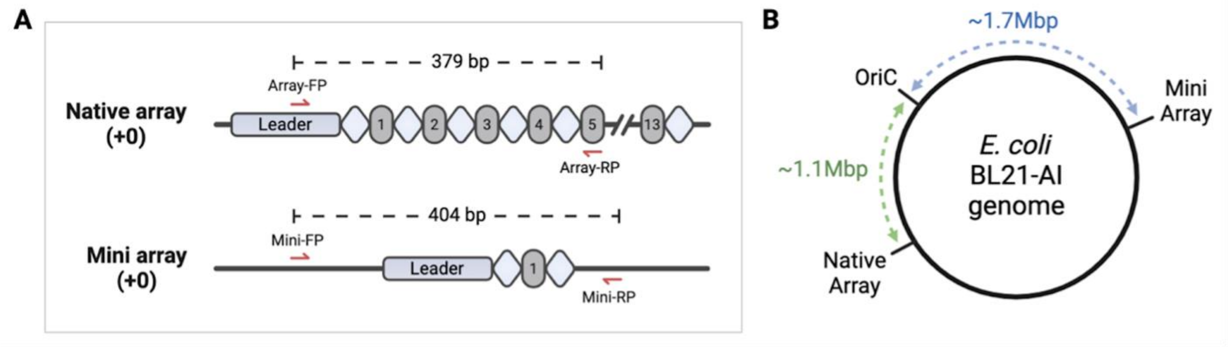

Supplementary Figure 12. Construction of an *E. coli* strain with two CRISPR arrays. (**A**) The *E. coli* BL21-AI host strain contains a native Type I-E CRISPR array. A second "mini" array was inserted into the genome. Primers for amplifying across the spacer integration sites are shown. (**B**) A map of the *E. coli* BL21-AI genome annotated with the two arrays and distances relative to the origin of replication (OriC).

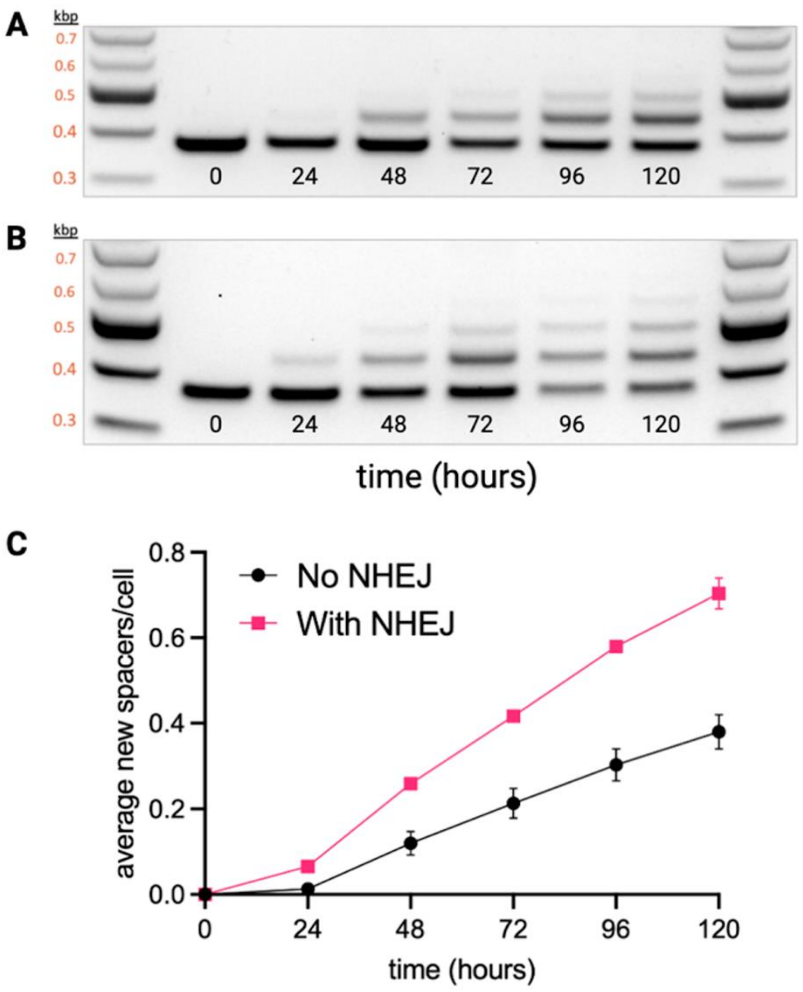

Supplementary Figure 13. NHEJ expression enhances spacer acquisition. (**A**) PCR products from a 5-day Cas1-Cas2 induction time course for the base recording strain containing pUC19. (**B**) The same strain as in A with the addition of NHEJ expression from an operon introduced into the genome. (**C**) Spacer acquisition from A and B plotted as average new spacers/cell in each culture. Means of three biological replicates ± SD are reported.

Supplemental Text 1. Genome integrated sequences

Cas1-Cas2:

taatacgactcactataggggaattgtgagcggataacaattcccctgtagaaataattttgtttaactttaataaggagatataccatgacctggcttc
cccttaatcccattccactcaaagatcgcgtctccatgatctttctgcaatatgggcagatcgatgtaatagatggcgcgtttgtacttatcgacaagac
agggatccgcactcatattcctgttggctcggttgcctgcatcatgctggaacctggtacacgggtttcgcatgcagctgtacgcctggctgcgcaa
gttggaacattgttggtatgggtgggggaagcgggcgttcgtgtttatgcttctggtcagcctggaggtgcgcgttcagataagctgctctatcagg
caaaacttgctctggatgaagatttgcgtctgaaggtcgtacgtaaaatgtttgaacttcggtttggagaacctgcgcctgcccggcgctccgtaga
gcaactcagaggtatagaaggcagtcgcgtgcgggcaacctacgcacttctggcgaagcaatacggcgtgacatggaatggacgtcgctacga
tccgaaagactgggaaaagggcgatacgatcaaccaatgcattagcgctgcaacttcctgtttatacggcgtaactgaagcggcgatacttgcagc
tggttatgcaccagctattgggtttgtgcatacaggaaagcctctttcctttgtttacgatattgcagacatcattaaatttgacactgttgtaccgaaagc

ttttgagatagcgcgtcgtaaccctggtgagccggaccgggaagtccgtttggcgtgcagggatatttttcgcagtagtaaaacattagccaaattg
attccgcttatagaggacgtgcttgccgctggagaaatacaaccgccggccccacctgaagatgcacagcctgttgccattccgcttcctgtttcac
tgggagatgcaggccatcggagtagctgaaatgagtatgttggtcgtggtcactgaaaatgtacctccgcgcttacgaggcagattagccatctgg
ttgttggaggtacgtgcaggggtatatgtaggtgatgtatccgcaaaaattcgtgaaatgatctgggaacaaatagctggactggcggaagaaggc
aatgtagtgatggcatgggcaacgaatacggaaacgggatttgagttccagacatttgggttaaacaggcgtaccccggtagatttggatggtttaa
ggttggtgtctttttttacctgtttgagcggccgcactcgagtctggtaaagaaaccgctgctgcgaaatttgaacgccagcacatggactcgtctact
agcgcagcttaattaacctaggctgctgccaccgctgagcaataactagcataaccccttggggcctctaaacgggtcttgaggggttttttg.

Cascade-Cas3:

tgttgatacaaccataaaatgataattacacccataaattgataattatcacacccataaattgatattgcctcttcatggtctaaacttcagtaagtttacg
acattttcctcgaggtcatttcctggatctggcctagttaatctttctgcgaattgagatgacgccactggctgggcgtcatcccggtttcccgggtaaa
caccaccgaaaaatagttactatcttcaaagccacattcggtcgaaatatcactgattaacaggcggctatgctggagaagatattgcgcatgacac
actctgacctgtcgcagatattgattgatggtcattccagtctgctggcgaaattgctgacgcaaaacgcgctcactgcacgatgcctcatcacaaaa
tttatccagcgcaaagggacttttcaggctagccgccagccgggtaatcagcttatccagcaacgtttcgctggatgttggcggcaacgaatcactg
gtgtaacgatggcgattcagcaacatcaccaactgcccgaacagcaactcagccatttcgttagcaaacggcacatgctgactactttcatgctcaa
gctgaccgataacctgccgcgcctgcgccatccccatgctacctaagcgccagtgtggttgccctgcgctggcgttaaatcccggaatcgccccc
tgccagtcaagattcagcttcagacgctccgggcaataaataatattctgcaaaaccagatcgttaacggaagcgtaggagtgtttatcgtcagcat
gaatgtaaaagagatcgccacgggtaatgcgataagggcgatcgttgagtacatgcaggccattaccgcgccagacaatcaccagctcacaaaa
atcatgtgtatgttcagcaaagacatcttgcggataacggtcagccacagcgactgcctgctggtcgctggcaaaaaaatcatctttgagaagtttta
actgatgcgccaccgtggctacctcggccagagaacgaagttgattattcgcaatatggcgtacaaatacgttgagaagattcgcgttattgcagaa
agccatcccgtccctggcgaatatcacgcggtgaccagttaaactctcggcgaaaaagcgtcgaaaagtggttactgtcgctgaatccacagcga
taggcgatgtcagtaacgctggcctcgctgtggcgtagcagatgtcgggctttcatcagtcgcaggcggttcaggtatcgctgaggcgtcagtccc
gtttgctgcttaagctgccgatgtagcgtacgcagtgaaagagaaaattgatccgccacggcatcccaattcacctcatcggcaaaatggtcctcca
gccaggccagaagcaagttgagacgtgatgcgctgttttccaggttctcctgcaaactgcttttacgcagcaagagcagtaattgcataaacaagat
ctcgcgactggcggtcgagggtaaatcattttccccttcctgctgttccatctgtgcaaccagctgtcgcacctgctgcaatacgctgtggttaacgc
gccagtgagacggatactgcccatccagctcttgtggcagcaactgattcagcccggcgagaaactgaaatcgatccggcgagcgatacagcac
attggtcagacacagattatcggtatgttcatacagatgccgatcatgatcgcgtacgaaacagaccgtgccaccggtgatggtatagggctgccc
attaaacacatgaatacccgtgccatgttcgacaatcacaatttcatgaaaatcatgatgatgttcaggaaaatccgcctgcgggagccggggttcta
tcgccacggacgcgttaccagacggaaaaaaatccacactatgtaatacggtcatactggcctcctgatgtcgtcaacacggcgaaatagtaatca
cgaggtcaggttcttaccttaaattttcgacggaaaaccacgtaaaaaacgtcgatttttcaagatacagcgtgaattttcaggaaatgcggtgagcat
cacatcaccacaattcagcaaattgtgaacatcatcacgttcatctttccctggttgccaatggcccattttcctgtcagtaacgagaaggtcgcgaatt
caggcgctttttagactggtcgtacgtatagaaatgcaggagaaatgtcggagcatatgaaggagaacaaatgaatttgcttattgataactggatcc
ctgtacgcccgcgaaacgggggggaaagtccaaatcataaatctgcaatcgctatactgcagtagagatcagtggcgattaagtttgccccgtgac
gatatggaactggccgctttagcactgctggtttgcattgggcaaattatcgccccggcaaaagatgacgttgaatttcgacatcgcataatgaatcc
gctcactgaagatgagtttcaacaactcatcgcgccgtggatagatatgttctaccttaatcacgcagaacatccctttatgcagaccaaaggtgtca
aagcaaatgatgtgactccaatggaaaaactgttggctggggtaagcggcgcgacgaattgtgcatttgtcaatcaaccggggcagggtgaagc
attatgtggtggatgcactgcgattgcgttattcaaccaggcgaatcaggcaccaggttttggtggtggttttaaaagcggtttacgtggaggaacac
ctgtaacaacgttcgtacgtgggatcgatcttcgttcaacggtgttactcaatgtcctcacattacctcgtcttcaaaaacaatttcctaatgaatcacat
acggaaaaccaacctacctggattaaacctatcaagtccaatgagtctatacctgcttcgtcaattgggtttgtccgtggtctattctggcaaccagcg
catattgaattatgcgatcccattgggattggtaaatgttcttgctgtggacaggaaagcaatttgcgttataccggttttcttaaggaaaaatttaccttt
acagttaatgggctatggccccatccgcattccccttgtctggtaacagtcaagaaaggggaggttgaggaaaaaatttcttgctttcaccacctccgc
accatcatggacacaaatcagccgagttgtggtagataagattattcaaaatgaaaatggaaatcgcgtggcggcggttgtgaatcaattcagaaat
attgcgccgcaaagtcctcttgaattgattatgggggatatcgtaataatcaagcatctattcttgaacggcgtcatgatgtgttgatgtttaatcagg
ggtggcaacaatacggcaatgtgataaacgaaatagtgactgttggtttgggatataaaacagccttacgcaaggcgttatatacctttgcagaagg

gtttaaaaataaagacttcaaaggggccggagtctctgttcatgagactgcagaaaggcatttctatcgacagagtgaattattaattcccgatgtact
ggcgaatgttaatttttcccaggctgatgaggtaatagctgatttacgagacaaacttcatcaattgtgtgaaatgctatttaatcaatctgtagctccct
atgcacatcatcctaaattaataagcacattagcgcttgcccgcgccacgctatacaaacatttacgggagttaaaaccgcaaggagggccatcaa
atggctgatgaaattgatgcaatggctttatatcgagcctggcaacaactggataatggatcatgtgcgcaaattagacgtgtttcagaacctgatga
attacgcgatatccctgcgttttataggctggtgcaaccttttggttgggaaaacccacgtcaccagcaggctcttttgcgcatggtgttttgcctgagc
gcaggaaagaatgtcatccgacatcaggacaaaaaatcggagcaaacaacaggtatctcgttgggaagagctttagccaatagtggaagaattaa
cgagcgccgtatctttcaattaattcgggctgacagaacagccgatatggtccagttacgtcgattacttactcacgccgaacccgtacttgactggc
cattaatggccaggatgttgacctggtggggaaagcgcgaacgccagcaacttctggaagattttgtattgaccacaaacaaaaatgcgtaagga
aacctttctatgtctaactttatcaatattcatgttctgatctctcacagcccttcatgtctgaaccgcgacgatatgaacatgcagaaagacgctattttc
ggcggcaaaagacgagtaagaatttcaagtcaaagccttaaacgtgcgatgcgtaaaagtggttattacgcacaaaatattggtgaatccagtctca
gaaccattcatcttgcacaattacgtgatgttcttcggcaaaaacttggtgaacgttttgaccaaaaaatcatcgataagacattagcgctgctctccg
gtaaatcagttgatgaagccgaaaagatttctgccgatgcggttactccctgggttgtgggagaaatagcctggttctgtgagcaggttgcaaaagc
agaggctgataatctggatgataaaaagctgctcaaagttcttaaggaagatattgccgccatacgtgtgaatttacagcagggtgttgatattgcgc
ttagtggaagaatggcaaccagcggcatgatgactgagttgggaaaagttgatggtgcaatgtccattgcgcatgcgatcactactcatcaggttga
ttctgatattgactggttcaccgctgtagatgatttacaggaacaaggttctgcacatctgggaactcaggaattttcatcgggtgtttttatcgttatgc
caacattaacctcgctcaacttcaggaaaatttaggtggtgcctccagggagcaggctctggaaattgcaacccatgttgttcatatgctggcaaca
gaggtccctggagcaaaacagcgtacttatgccgcttttaaccctgcggatatggtaatggttaatttctccgatatgccactttctatggcaaatgctt
ttgaaaaagcggttaaagcgaaagatggctttttgcaaccgtctatacaggcgtttaatcaatattgggatcgcgttgccaatggatatggtctgaac
ggagctgctgcgcaattcagcttatctgatgtagacccaattactgctcaagttaaacaaatgcctactttagaacagttaaaatcctgggttcgtaata
atggcgaggcgtgaacatgagatcttatttgatcttgcggcttgctgggccaatgcaagcctgggggcagccgacctttgaaggaacgcgaccta
ccggaagatttccgacccgaagcgggttattagggctactcggggcttgtcttgggatccaacgtgatgatacttcttcattacaggcgttatcagag
agtgtgcaatttgcagtgcgctgcgatgaactcattcttgacgatcgtcgtgtgtctgtaacggggttgcgtgattaccatacagtccttggagcgcg
agaagattaccgtggtttgaaaagtcatgaaacgattcaaacatggcgcgaatatttatgtgatgcctcctttaccgtcgctctctggttaacaccccat
gcaacgatggttatctcagaacttgaaaaagcagtattaaagcctcggtatacaccttacctggggcggagaagttgcccactaacacacccgcttt
ttttggggacatgtcaggcatcggatcctcagaaggcgctattaaattatgagcccgttggcggcgatatatatagtgaggaatcagttacagggca
tcatttaaaatttacggcgcgcgacgaaccgatgatcaccttgcctcgacaatttgcttcccgagaatggtatgtgattaaaggaggtatggatgtatc
tcagtaaagtcatcattgccagggcctggagcagggatctttaccaacttcaccagggattatggcatttatttccaaacagaccggatgctgctcgt
gattttctttttcatgttgagaagcgaaacacaccagaaggctgtcatgttttattgcagtcagcgcaaatgcctgtttcaactgccgttgcgacagtca
ttaaaactaaacaggttgaatttcaacttcaggttggtgttccactctattttcggcttcgggcaaatccgatcaaaactattctcgacaatcaaaagcg
cctggacagtaaagggaatattaaacgctgtcgggttccgttaataaaagaagcagaacaaatcgcgtggttgcaacgtaaattgggcaatgcgg
cgcgcgttgaagatgtgcatcccatatcggaacggccacagtatttttctggtgatggtaaaagtggaaagatccaaacggtttgctttgaaggtgtg
ctcaccatcaacgacgcgccagcgttaatagatcttgtacagcaaggtattgggccagctaaatcgatgggatgtggcttgctatctttggctccact
gtgagggaggctattaatggaaccttttaaatatatatgccattactggggaaaatcctcaaaaagcttgacgaaaggaaatgatattcatctgttaatt
tatcattgccttgatgttgctgctgttgcagattgctggtgggatcaatcagtcgtactgcaaaatactttttgccgaaatgaaatgctatcaaaacaga
gggtgaaggcctggctgttatttttcattgctcttcatgatattggaaagtttgatatacgattccaatataaatcagcagaaagttggctgaaattaaatc
ctgcaacgccatcacttaatggtccatcaacacaaatgtgccgtaaatttaatcatggtgcagccggtctgtattggtttaaccaggattcactttcaga
gcaatctctcggggatttttcagtttttttgatgccgctcctcatccttatgagtcctggtttccatgggtagaggccgttacaggacatcatggttttata
ttacattcccaggatcaagataagtcgcgttgggaaatgccagcttctctggcatcttatgctgcgcaagataaacaggctcgtgaggagtggatat
ctgtactggaagcattatttttaacgccagcggggttatctataaacgatataccacctgattgttcatcactgttagcaggtttttgctcgcttgctgact
ggttaggctcctggactacaacgaatacctttctgtttaatgaggatgcgccttccgacataaatgctctgagaacgtatttccaggaccgacagcag
gatgcgagccgggtattggagttgagtggacttgtatcaaataagcgatgttatgaaggtgttcatgcactactggacaatggctatcaacccagac
aattacaggtgttagttgatgctcttccagtagctcccgggctgacggtaatagaggcacctacaggctccggtaaaacggaaacagcgctggcct
atgcttggaaacttattgatcaacaaattgcggatagtgttatttttgccctcccaacacaagctaccgcgaatgctatgcttacgagaatggaagcga
gcgcgagccacttattttcatccccaaatcttattcttgctcatggcaattcacggtttaaccacctctttcaatcaataaaatcacgcgcgattactgaa
caggggcaagaagaagcgtgggttcagtgttgtcagtggttgtcacaaagcaataagaaagtgtttcttgggcaaatcggcgtttgcacgattgatc
aggtgttgatatcggtattgccagttaaacaccgctttatccgtggtttgggaattggtcgaagtgttttaattgttgatgaagttcatgcttacgacacct
atatgaacggcttgctggaggcagtgctcaaggctcaggctgatgtgggagggagtgttattcttctttccgcaaccctaccaatgaaacaaaaaca
gaaacttctggatacttatggtctgcatacagatccagtggaaaataactccgcatatccactcattaactggcgaggtgtgaatggtgcgcaacgtt

ttgatctgctagctcatccagaacaactcccgccccgcttttcgattcagccagaacctatttgtttagctgacatgttacctgaccttacgatgttagag
cgaatgatcgcagcggcaaacgcgggtgcacaggtctgtcttatttgcaatttggttgacgttgcacaagtatgctaccaacggctaaaggagcta
aataacacgcaagtagatatagatttgtttcatgcgcgctttacgctgaacgatcgtcgtgaaaaagagaatcgagttattagcaatttcggcaaaaat
gggaagcgaaatgttggacggatacttgtcgcaacccaggtcgtggaacaatcactcgacgttgattttgattggttaattactcagcattgtcctgc
agatttgcttttccaacgattgggccgtttacatcgccatcatcgcaaatatcgtcccgctggttttgagattcctgttgccaccattttgctgcctgatgg
cgagggttacggacgacatgagcatatttatagcaacgttagagtcatgtggcggacgcagcaacatattgaggagcttaatggagcatccttatttt
tccctgatgcttaccggcaatggctggatagcatttacgatgatgcggaaatggatgagccagaatgggtcggcaatggcatggataaatttgaaa
gcgccgagtgtgaaaaaaggttcaaggctcgcaaggtcctgcagtgggctgaagaatatagcttgcaggataacgatgaaaccattcttgcggta
acgagggatggggaaatgagcctgccattattgccttatgtacaaacgtcttcaggtaaacaactgctcgatggccaggtctacgaggacctaagt
catgaacagcagtatgaggcgcttgcacttaatcgcgtcaatgtacccttcacctggaaacgtagtttttctgaagtagtagatgaagatgggttactt
tggctggaagggaaacagaatctggatggatgggtctggcagggtaacagtattgttattacctatacaggggatgaagggatgaccagagtcat
ccctgcaaatcccaaataacttcgggaatgattgttatcaatgacgataataagaccaataacggtttatccctacttaagtagggaaggtgcacaat
gtacaccttctagagtccttactgcagtagttttgctgaaatactcgattcacaaaaatatcaacttatggttgttttgtgagatatcaatatatggttgtttt
gtggttaagttgctgattataaataattattaaatatcactttatggttgcatcaaca.

Ku-LigD (NHEJ):

tgttgatacaaccataaaatgataattacacccataaattgataattatcacacccataaattgatattgcctcttcatggtctaaacttcagtaagtttacg
acattttcctcgaggtcatttccaacctttgcggtatggcatgatagcgcgacgtcttaagacccactttcacatttaagttgtttttctaatccgcatatg
atcaattcaaggccgaataagaaggctggctctgcaccttggtgatcaaataattcgatagcttgtcgtaataatggcggcatactatcagtagtagg
tgtttccctttcttctttagcgacttgatgctcttgatcttccaatacgcaacctaaagtaaaatgccccacagcgctgagtgcatataatgcattctctag
tgaaaaaccttgttggcataaaaaggctaattgattttcgagagtttcatactgtttttctgtaggccgtgtacctaaatgtacttttgctccatcgcgatg
acttagtaaagcacatctaaaacttttagcgttattacgtaaaaaatcttgccagctttccccttctaaagggcaaaagtgagtatggtgcctatctaac
atctcaatggctaaggcgtcgagcaaagcccgcttattttttacatgccaatacaatgtaggctgctctacacctagcttctgggcgagtttacgggtt
gttaaaccttcgattccgacctcattaagcagctctaatgcgctgttaatcactttacttttatctaatctagacatcattaattcctaatttttgttgacactct
atcgttgatagagttattttaccactccctatcagtgatagagaaaagaattcaaaagatctaaagaggagaaaggatctatgcgcagcatttggaaa
ggcagcattgcgtttggcctggtgaacgtgccggtgaaagtgtatagcgcgaccgaagatcatgatattaaatttcatcaggtgcatgcgaaagata
acggccgcattcgctataaacgcgtgtgcgaagtgtgcggcgaagtggtggaatatcgcgatattaacaaagcgtttgaaagcgatgatggccag
atggtggtgattaccgatgaagatattgcgaccctgccggaagaacgcagccgcgaaattgaagtggtggaatttattccggcggaacagctgga
tccgctgatgtatgataaaagctattttctggaaccggatagcaaaagcagcaaaagctatgtgctgctggcgaaaaccctggcggaaaccgatcg
cattgcgattgtgcattttagcctgcgcaacaaaagccgcctggcggcgctgcgcgtgaaagattttagcaaacgcgatgtgatgatgattcatacc
ctgctgtggccggatgaaattcgcgatccggattttccgattctggataaagaagtgcagattaaaccggcggaactgaaaatggcgggccaggt
ggtggaaagcatgaccgatgattttaaaccggatctgtatcatgatgattatcaggaacagctgcgcgaactggtgcaggcgaaactggaaggcg
gcgaagcgtttagcgtggaagaacagccggcggaactggatgaaggcaccgaagatgtgagcgatctgctggcgaaactggaagcgagcgtg
aaagcgcgcaaaggcggcaaaagcgatagcaaagatgatagcgatagcgaaagcgatagcaaagaaagcaaaagcgatagcaaaccggcg
aaaaaagcgccggcgaaaaaagcggcggcgaaaaaaagcaccgcgaaaaaagcgccggcgaaaaaagcggcggcgaaaaaaagctgaa
atggcgcgccatccgtggggcatggaacgctatgaacgcgtgcgcctgaccaacccggataaagtgctgtatccggcgaccggcaccaccaaa
gcggaagtgtttgattattatctgagcattgcgcaggtgatggtgccgcatattgcgggccgcccggtgacccgcaaacgctggccgaacggcgt
ggcggaagaagcgtttttgaaaaacagctggcgagcagcgcgccgagctggctggaacgcggcagcattacccataaaagcggcaccacca
cctatccgattattaacacccgcgaaggcctggcgtgggtggcgcagcaggcgagcctggaagtgcatgtgccgcagtggcgctttgaagatgg
cgatcagggcccggcgacccgcattgtgtttgatctggatccgggcgaaggcgtgaccatgacccagctgtgcgaaattgcgcatgaagtgcgc
gcgctgatgaccgatctggatctggaaacctatccgctgaccagcggcagcaaaggcctgcatctgtatgtgccgctggcggaaccgattagca
gccgcggcgcgagcgtgctggcgcgccgcgtggcgcagcagctggaacaggcgatgccgaaactggtgaccgcgaccatgaccaaaagcc
tgcgcgcgggcaaagtgtttctggattggagccagaacaacgcggcgaaaaccaccattgcgccgtatagcctgcgcggccgcgatcatccga
ccgtggcggcgccgcgcacctgggatgaaattgcggatccggaactgcgccatctgcgctttgatgaagtgctggatcgcctggatgaatatggc

gatctgctggcgccgctggatgcggatgcgccgattgcggataaactgaccacctatcgcagcatgcgcgatgcgagcaaaaccccggaaccg
gtgccgaaagaaattccgaaaaccggcaacaacgataaatttgtgattcaggaacatcatgcgcgccgcctgcattatgatctgcgcctggaacg
cgatggcgtgctggtgagctttgcggtgccgaaaaacctgccggaaaccaccgcggaaaaccgcctggcggtgcataccgaagatcatccgatt
gaatatctggcgtttcatggcagcattccgaaaggcgaatatggcgcgggcgatatggtgatttgggatagcggcagctatgaaaccgaaaaattt
cgcgtgccggaagaactggataacccggatgatagccatggcgaaattattgtgaccctgcatggcgaaaaagtggatggccgctatgcgctgat
tcagaccaaaggcaaaaactggctggcgcatcgcatgaaagatcagaaaaacgcgcgcccggaagattttgcgccgatgctggcgaccgaag
gcagcgtggcgaaatataaagcgaaacagtgggcgtttgaaggcaaatgggatggctatcgcgtgattattgatgcggatcatggccagctgcag
attcgcagccgcaccggccgcgaagtgaccggcgaatatccgcagtttaaagcgctggcggcggatctggcggaacatcatgtggtgctggat
ggcgaagcggtggcgctggatgaaagcggcgtgccgagctttggccagatgcagaaccgcgcgcgcagcacccgcgtggaattttgggcgttt
gatattctgtggctggatggccgcagcctgctgcgcgcgaaatatagcgatcgccgcaaaattctggaagcgctggcggatggcggcggcctga
ttgtgccggatcagctgccgggcgatggcccggaagcgatggaacatgtgcgcaaaaaacgctttgaaggcgtggtggcgaaaaaatgggata
gcacctatcagccgggccgccgcagcagcagctggattaaagataaaatttggaacacccaggaagtggtgattggcggctggcgccagggc
gaaggcggccgcagcagcggcattggcgcgctggtgctgggcattccgggcccggaaggcctgcagtttgtgggccgcgtgggcaccggctt
taccgaaaaagaactgagcaaactgaaagatatgctgaaaccgctgcataccgatgaaagcccgtttaacgcgccgctgccgaaagtggatgcg
cgcggcgtgacctttgtgcgcccggaactggtgggcgaagtgcgctatagcgaacgcaccagcgatggccgcctgcgccagccgagctggcg
cggcctgcgcccggataaaaccccggatgaagtggtgtgggaatgattaacctaggctgctgccaccgctgagcaataaacactcaggcatttga
gaagcacacggtcactccggcaattaaaaaagcggctaaccacgccgctttttttacgtctgcactcggtaccaaattccagaaaagaggcctccc
gaaagggggccttttttcgttttggtccccggtaaaccagcaatagacgctagtaccagttgagcaaggccgaactcagaagtgaaaccttctag
agtccttactgcagtagttttgctgaaatactcgattcacaaaaatatcaacttatggttgttttgtgagatatcaatatatggttgttttgtggttaagttgct
gattataaataattattaaatatcactttatggttgcatcaaca.

Mini CRISPR array

Tgttgatacaaccataaaatgataattacacccataaattgataattatcacacccataaattgatattgcctcttcatggtctaaacttcagtaagtttac
gacattttcctcgaggtcatttccttggaatttacagcgaggcggtttatccccgctggcgcggggaacacccgtaagtggtttgagcgatgatattt
gtgctcggtttatccccgctggcgcggggaacactctaaacataacctattattaattaatgattttttaagccagtcacaatctaccaactttatagtatc
acacaaacaacacatccattatgttaaagagtacttaatccattgattaaaaaggtaaatatttaaaataactctatacaaactaaaatctaccaaacttt
accgcaataattttcactccagcgaaaaattaatgccacagaatttgtagagaatgctaatgattagccctggatgggtaaccttctagagtccttact
gcagtagttttgctgaaatactcgattcacaaaaatatcaacttatggttgttttgtgagatatcaatatatggttgttttgtggttaagttgctgattataaat
aattattaaatatcactttatggttgcatcaaca.

Supplemental Text 2. Modeling array expansion with variable growth in the two-array strain

In the case of the strain containing both the native array and mini array, the number of cells with a given array length at a given time is determined similarly to Equations 3 and 4 of the main text. Shown below is the system of equations representing the number of cells with each native array length.

$$\frac{dN_0^{nat}}{dt} = \mu_0^{nat} N_0^{nat} - r^{nat} N_0^{nat}$$

$$\frac{dN_i^{nat}}{dt} = (\mu_i^{nat} - r^{nat}) N_i^{nat} + r^{nat} N_{i-1}^{nat}$$

The key difference arises in the determination of the growth rates associated with each array length. In the main body, a variable growth rate was described using the form $\mu_i = \mu_0 \alpha^i$. Because each cell contains both arrays, cells with any given native array length contain a representative population of mini array lengths, and vice versa. Accordingly, growth at any given native or mini array length is determined via a weighted sum of the growth rates of the other array. Shown below for unexpanded native arrays,

$$\mu_0^{nat} = \mu_0(F_0^{mini} + \alpha F_1^{mini} + \alpha^2 F_2^{mini} + \cdots)$$

where

$$F_j^{mini} = \frac{N_j^{mini}}{\sum_j N_j^{mini}}$$

Generalizing to any native array length yields the following expression:

$$\mu_i^{nat} = \mu_i \left(\sum_j F_j^{mini} \alpha^j \right)$$

Plugging in our earlier definition of μ_i, we can rewrite the previous expression as:

$$\mu_i^{nat} = \mu_0 \left(\sum_j F_j^{mini} \alpha^{i+j} \right)$$

This representation also describes the growth rates for each mini array length population by swapping the tags ‘mini’ and ‘nat’. These coupled equations, assuming a single μ_0 and α, were used to determine the expansion rates presented in Fig. 3d of the main body.

Supplemental Text 3. Calculating array expansion rate in Figure 2C

Let N_i be the number of cells with i spacers in the population. N_i changes with respect to time according to:

$$\frac{dN_0(t)}{dt} = N_0(t)\left(\mu - R_{f,0}\right)$$

$$\frac{dN_i(t)}{dt} = N_i(t)\left(\mu - R_{f,i}\right) + N_{i-1}(t)\, R_{f,i-1}$$

Where cells at all array lengths have the same growth rate constant μ and acquisition rate R_f. Starting with an unexpanded cell population of M_0, solving this set of differential equations gives:

$$N_0(t) = M_0 e^{(\mu - R_f)t}$$

$$N_i(t) = \frac{1}{i!} M_0 \left(R_f t\right)^i e^{(\mu - R_f)t}$$

The fraction of cells at array length *i* within the population is therefore:

$$f_i(t) = \frac{N_i(t)}{\sum_{j=0}^{\infty} N_j(t)} = \frac{\frac{1}{i!} M_0 \left(R_f t\right)^i e^{(\mu - R_f)t}}{\sum_{j=0}^{\infty} \frac{1}{j!} M_0 \left(R_f t\right)^j e^{(\mu - R_f)t}}$$

Cancelling M_0 and $e^{(\mu - R_f)t}$, the fraction simplifies to:

$$f_i(t) = \frac{\frac{1}{i!} \left(R_f t\right)^i}{\sum_{j=0}^{\infty} \frac{1}{j!} \left(R_f t\right)^j}$$

showing that under the assumption where cells of different array lengths all grow at the same rate μ, the fractions of cells of each array length in the population over time depend only on the acquisition rate. Furthermore, using the +0 fraction and making a substitution in the denominator:

$$f_0(t) = e^{-R_f t}$$

it can be shown that the acquisition rate *r* equals the exponential decay rate of $f_0(t)$.
